# Lack of Usefulness of Literature‐Based *Helicobacter pylori* Resistance Data for Predicting First‐Line Empirical Treatment Effectiveness in the European Registry on *H. pylori* Management (Hp‐EuReg)

**DOI:** 10.1111/hel.70169

**Published:** 2026-11-05

**Authors:** Ismail Kirrout, Olga P. Nyssen, Ángeles Pérez‐Aísa, Bojan Tepes, Irina N. Voynovan, Samuel J. Martínez‐Domínguez, Alfredo J. Lucendo, Ludmila G. Vologzanina, Matteo Pavoni, Sayar R. Abdulkhakov, Maja Denkovski, Javier Tejedor‐Tejada, Tatiana Ilchishina, Luis Bujanda, Aiman S. Sarsenbaeva, Jose M. Huguet, Oleg Zaytsev, Boris D. Starostin, Monica Perona, Antonietta G. Gravina, Manuel Pabón‐Carrasco, Sergey A. Alekseenko, Óscar Núñez, Miguel Areia, Jesús Barrio, Antonio Moreno Loro, Dmitry N. Andreev, Blas José Gómez Rodríguez, María Soledad Marcos, Inmaculada Ortiz‐Polo, Galina N. Tarasova, Igor Bakulin, Mónica Sánchez Alonso, Irene Arteagoitia, Virginia Flores, Manuel Jiménez‐Moreno, Dmitry S. Bordin, Antonio Gasbarrini, Ricardo Marcos‐Pinto, Wojciech Marlicz, Perminder S. Phull, Marino Venerito, Doron Boltin, Lyudmila Boyanova, Pablo Parra, Leticia Moreira, Francis Mégraud, Colm O'Morain, Nuria Montes, Javier P. Gisbert, the Hp‐EuReg investigators, Natalia N. Dekhnich, Pilar Mata‐Romero, Natalia Valerievna Bakulina, Alla G. Kononova, Giuseppe Losurdo, Judith Gomez‐Camarero, Alma Keco‐Huerga, Pablo M. Wolfe García, Jurij Bednarik, Eduardo Iyo, Antonia Perelló, Luis Fernández‐Salazar, Noelia Alcaide, Benito Velayos, Pedro Almela, Ramón Pajares Villarroya, Daniel Martin‐Holgado, Miguel Fernández‐Bermejo, Ian L. P. Beales, Alisan Kahraman, Ana Beatriz Pozo Blanco, Montserrat Planella, Mikel Ganuza, Victor A. Kamburov, Marina F. Osipenko, Debora Compare, Georges Kamtoh, Rosa Rosania, Eva Barreiro Alonso, Maria A. Livzan, Teresa Angueira, Consuelo Ramírez, Piotr Szredzki, Natalia Baryshnikova, Natalia Bakanova, Pedro Delgado Guillena, Sheyla Montori Pina, Eduardo Albéniz, Piotr Eder, Inna V. Savilova, Henrique Fernandes‐Mendes, Miguel Suárez Matías, María Badía Martínez, Liya Nikolaevna Belousova, Benito Hermida Pérez, Ana Garre, Natasa Brglez Jurecic, Olga Kolokolnikova, Paola Chaudarcas, Luis Hernández, Regina I. Khlynova, Antonio Cuadrado, Marta Pascual‐Mato, Ekaterina Y. Plotnikova, Mila Kovacheva‐Slavova, Ramiro Carreño Macián, Manon C. W. Spaander, Guillem Soy, Pilar Pazo Mejide, Sergio Gil Rojas, Carlos Maroto‐Martín, Francisco J. Rancel‐Medina, Diego Burgos‐Santamaría, Isabel Pérez‐Martínez, Giulia Fiorini, Teresa Valdés‐Lacasa, Riccardo Vasapolli, Rosa M. Sáiz‐Chumillas, Tamara Matysiak‐Budnik, Anna L. Pakhomova, Jorge Yebra Carmona, Diego Ledro Cano, Senador Moran Sanchez, Jan Bornschein, Pilar Bernal Checa, Antonio M. Caballero‐Mateos, Leticia Gimeno Pitarch, Ángela Martínez Herreros, Nerea Alonso‐Bilbao, Patricia Sanz‐Segura, Jose Xavier Segarra Ortega, Raquel García‐Sánchez, Alexander Link, Jesus M. Gonzalez‐Santiago, Laura Larrey Ruiz, Rodrigo Garcés‐Durán

**Affiliations:** ^1^ Methodology Unit, Instituto de Investigación Sanitaria Princesa (IIS‐Princesa), Rheumatology Service Hospital Universitario de La Princesa Madrid Spain; ^2^ Centro de Biotecnología y Genómica de Plantas UPM‐INIA/CSIC (CBGP) Universidad Politécnica de Madrid Madrid Spain; ^3^ Department of Gastroenterology Hospital Universitario de La Princesa, Instituto de Investigación Sanitaria Princesa (IIS‐Princesa), Universidad Autónoma de Madrid (UAM), and Centro de Investigación Biomédica en Red de Enfermedades Hepáticas y Digestivas (CIBEREHD) Madrid Spain; ^4^ Department of Gastroenterology Hospital Universitario Costa del Sol, RICAPPS–Red de Investigación en Cronicidad, Atención Primaria y Prevención y Promoción de la Salud Marbella Spain; ^5^ Department of Gastroenterology DC Rogaska Rogaska Slatina Slovenia; ^6^ A.S. Loginov Moscow Clinical Scientific Center Moscow Russia; ^7^ Department of Gastroenterology Hospital Clínico Universitario Lozano Blesa, Instituto de Investigación Sanitaria de Aragón (IIS Aragón), Centro de Investigación Biomédica en Red de Enfermedades Hepáticas y Digestivas (CIBEREHD) Zaragoza Spain; ^8^ Department of Gastroenterology Hospital General de Tomelloso, Instituto de Investigación Sanitaria Princesa (IIS‐Princesa), Centro de Investigación Biomédica en Red de Enfermedades Hepáticas y Digestivas (CIBEREHD), Instituto de Investigación Sanitaria de Castilla‐La Mancha (IDISCAM) Tomelloso Spain; ^9^ Gastrocentr Perm Russia; ^10^ Department of Medical and Surgical Sciences Sant'orsola‐Malpighi University Hospital Bologna Italy; ^11^ Department of Internal Diseases Institute of Fundamental Medicine and Biology, Kazan (Volga Region) Federal University Kazan Russia; ^12^ Department of Primary Care and General Practice Kazan State Medical University Kazan Russia; ^13^ Interni Oddelek Diagnostic Centre Bled Slovenia; ^14^ Department of Gastroenterology Hospital Universitario de Cabueñes Gijón Asturias Spain; ^15^ Department of Gastroenterology SM‐Clinic Saint‐Petersburg Russia; ^16^ Department of Gastroenterology, Biodonostia Health Research Institute, Department of Medicine Universidad del País Vasco (UPV/EHU), Centro de Investigación Biomédica en Red de Enfermedades Hepáticas y Digestivas (CIBEREHD) San Sebastián Spain; ^17^ Department of Gastroenterology Chelyabinsk Regional Clinical Hospital Chelyabinsk Russia; ^18^ Department of Gastroenterology Hospital General Universitario de Valencia Valencia Spain; ^19^ First Clinical Medical Centre Kovrov Russia; ^20^ Saint‐Petersburg State Budgetary Institution Healthcare City Policlinic 38 Saint‐Petersburg Russia; ^21^ Department of Gastroenterology Hospital Quirón Marbella Marbella Spain; ^22^ Department of Hepatogastroenterology University of Study of Campania “L. Vanvitelli” Naples Italy; ^23^ Department of Gastroenterology Hospital Universitario Virgen de Valme Sevilla Spain; ^24^ Far Eastern State Medical University Khabarovsk Russia; ^25^ Department of Gastroenterology Hospital Universitario Sanitas La Moraleja Madrid Spain; ^26^ Faculty of Medicine Universidad CEU San Pablo Madrid Spain; ^27^ Department of Gastroenterology Portuguese Oncology Institute of Coimbra, RISE@CI‐IPO (Health Research Network), Portuguese Oncology Institute of Porto (IPO Porto) Coimbra Portugal; ^28^ Department of Gastroenterology Hospital Universitario Río Hortega, Gerencia Regional de Salud de Castilla y León (SACYL) Valladolid Spain; ^29^ Department of Gastroenterology Hospital Universitario Virgen del Rocío Sevilla Spain; ^30^ Russian University of Medicine Moscow Russia; ^31^ Department of Gastroenterology Hospital Universitario Virgen Macarena Sevilla Spain; ^32^ Department of Gastroenterology Hospital 12 de Octubre Madrid Spain; ^33^ Department of Gastroenterology Hospital Universitario y Politécnico la Fe Valencia Spain; ^34^ Department of Gastroenterology Rostov State Medical University Rostov‐on‐Don Russia; ^35^ II Mechnikov North‐Western State Medical University Saint Petersburg Russia; ^36^ Department of Gastroenterology Hospital Universitario Santa Bárbara Ciudad Real Spain; ^37^ Department of Gastroenterology Hospital Universitario de Cruces Vizcaya Spain; ^38^ Department of Gastroenterology Hospital General Universitario Gregorio Marañón Madrid Spain; ^39^ Department of Gastroenterology Hospital Universitario de Burgos Burgos Spain; ^40^ Department of Pancreatic Biliary and Upper Digestive Tract Disorders, A. S. Loginov Moscow Clinical Scientific Center Moscow Russia; ^41^ Department of Outpatient Therapy and Family Medicine Tver State Medical University Tver Russia; ^42^ Department of Propaedeutic of Internal Diseases and Gastroenterology Russian University of Medicine Moscow Russia; ^43^ Department of Internal Medicine and Gastroenterology Fondazione Policlinico Universitario Agostino Gemelli IRCCS, Università Cattolica del Sacro Cuore Rome Italy; ^44^ Department of Gastroenterology Centro Hospitalar Do Porto, Instituto De Ciências Biomédicas de Abel Salazar, Universidade do Porto, Center for Research in Health Technologies and Information Systems (CINTESIS) Porto Portugal; ^45^ Department of Gastroenterology Pomeranian Medical University in Szczecin, the Centre for Digestive Diseases Szczecin Poland; ^46^ Department of Digestive Disorders Aberdeen Royal Infirmary Aberdeen UK; ^47^ Department of Gastroenterology, Hepatology and Infectious Diseases Otto‐von‐Guericke University Hospital Magdeburg Germany; ^48^ Department of Gastroenterology Rabin Medical Center, Tel Aviv University Tel Aviv Israel; ^49^ Department of Medical Microbiology Medical University of Sofia Sofia Bulgaria; ^50^ Department of Gastroenterology Hospital Clínic de Barcelona, Centro de Investigación Biomédica en Red en Enfermedades Hepáticas y Digestivas (CIBEREHD), Institut D'investigacions Biomèdiques August Pi i Sunyer (IDIBAPS), University of Barcelona Barcelona Spain; ^51^ INSERM U1312 BRIC, Université de Bordeaux Bordeaux France; ^52^ Faculty of Health Sciences Trinity College Dublin Dublin Ireland

**Keywords:** antibiotic resistance, eradication, *Helicobacter pylori*, literature, registry, treatment

## Abstract

**Background:**

*Helicobacter pylori* ( *H. pylori* ) infection causes gastritis, peptic ulcer disease, and gastric cancer. Without an effective vaccine, eradication therapy remains crucial. However, increasing antibiotic resistance compromises treatment effectiveness.

**Objective:**

To evaluate whether literature‐based resistance data could predict first‐line empirical treatment effectiveness.

**Methods:**

Resistance rates for clarithromycin, metronidazole, and levofloxacin were extracted from bibliographic reviews and estimated for three timeframes: the treatment year, the preceding year, and the last five years average. These rates (2008–2024) were matched with outcomes from the European Registry on *H. pylori* Management (Hp‐EuReg, WorldHpReg partner), a prospective real‐world study involving 40 countries. Analyses included Spearman's correlations, multivariate logistic regressions adjusted for demographic, clinical, and therapeutic‐related variables, bootstrap resampling stratified by eradication success, and Random Forest models.

**Results:**

Analysis included 37,659 patients receiving first‐line empirical treatment from 13 countries. Moderate negative correlations were observed between metronidazole and clarithromycin resistances for some regimens. In the regression model, including either contemporaneous or previous‐year resistance rates, clarithromycin resistance showed a negligible positive association, while metronidazole resistance was negatively associated. In regimen‐specific models, previous‐year clarithromycin resistance was positively associated with success in concomitant therapy, whereas higher five‐year mean clarithromycin resistance reduced eradication odds in quadruple clarithromycin–amoxicillin–bismuth therapy. Random Forest models identified compliance, proton‐pump inhibitor dose, regimen duration, and treatment as the strongest predictors of eradication, while resistance variables contributed minimally.

**Conclusion:**

Literature‐based resistance data are not useful for guiding real‐world first‐line *H. pylori* therapy, highlighting the need for individualized antibiotic susceptibility testing to obtain reliable resistance information.

**Trial Registration:**

ClinicalTrials.gov identifier: NCT02328131

## 1 Introduction

*Helicobacter pylori* ( *H. pylori* ) infects approximately half of the global population and is the main cause of chronic gastritis, peptic ulcer disease, gastric adenocarcinoma, and gastric mucosa‐associated lymphoid tissue lymphoma. In the absence of an effective vaccine, management continues to rely on antibiotic‐based eradication therapy [1, 2]. However, the effectiveness of standard clarithromycin‐based triple therapy has declined, largely because of increasing antimicrobial resistance to this antibiotic [3], although poor adherence, insufficient treatment duration, and inadequate acid suppression also contribute to failure [4].

Resistance to clarithromycin, metronidazole, and levofloxacin now exceeds 15% in most World Health Organization regions and remains particularly high in Europe [5, 6, 7]. Accordingly, international guidelines recommend avoiding clarithromycin‐based triple therapy when resistance exceeds 15% or is unknown, favoring bismuth‐containing quadruple therapy and reserving levofloxacin‐based regimens mainly for rescue treatment [1, 8].

However, implementation remains challenging [9] and, while susceptibility‐guided therapy may improve eradication outcomes, testing is not routinely available [5, 10] and empirical treatment often depends on published resistance estimates or regional surveillance data. Whether these aggregated estimates reliably predict real‐world treatment success remains uncertain.

Therefore, the aim of this study was to assess the value of literature‐derived resistance rates for guiding treatment, by matching these resistance rates with first‐line empirical eradication outcomes from the European Registry on *H. pylori* Management (Hp‐EuReg).

## 2 Materials and Methods

### 2.1 Data Collection and Management

#### 2.1.1 Data From the Literature

Antibiotic resistance rates for clarithromycin, metronidazole, and levofloxacin were obtained from a recent systematic review performed by the authors [7]. For each country‐year pair, resistance prevalence was calculated as the mean of study‐reported percentages of resistant isolates among tested cases. Three timeframes were examined: same‐year resistance (estimate for the country and calendar year of treatment), prior‐year resistance (estimate from the preceding year, as an approximation to the data available at prescribing time), and 5‐year average resistance (average of the treatment year and the 4 preceding years, capturing a broader background resistance while smoothing short‐term fluctuations). These estimates were linked to first‐line empirical treatment effectiveness in Hp‐EuReg, assessed by a modified intention‐to‐treat (mITT) approach, which included all patients with a recorded first‐line regimen and confirmed follow‐up outcome regardless of compliance.

#### 2.1.2 Data From Real Clinical Practice

Hp‐EuReg is an ongoing international, multicentre, prospective, non‐interventional study, promoted by the European Helicobacter and Microbiota Study Group (www.helicobacter.org) [11]. Since its launch in 2013, Hp‐EuReg has collected real‐world clinical data from over 400 investigators across 40 countries. Hp‐EuReg includes anonymised patient‐level information on treatments, eradication and safety outcomes recorded in an e‐CRF via the REDCap platform [12] hosted by the “Asociación Española de Gastroenterología” (AEG; www.aegastro.es), a non‐profit Scientific and Medical Society focused on Gastroenterology research.

The study protocol complied with the last revision of ethical guidelines of the Declaration of Helsinki [13] and was approved by the institutional ethics committee. It was classified by the Spanish Agency for Medicines and Health Products, prospectively registered at ClinicalTrials.gov (NCT02328131), and received approval in 2012 from the Ethics Committee of Hospital Universitario de La Princesa, the reference Institutional Review Board. The protocol publication outlines criteria for country selection, national coordinators, and recruiting gastroenterologists [11].

### 2.2 Data Synthesis

For this analysis, the most frequent first‐line empirical regimens from Hp‐EuReg were matched by country and treatment year to the literature‐derived resistance estimates. Only countries with patients for whom a resistance estimate was available for the predefined timeframes were included. Consequently, the final dataset comprised matched registry and resistance data from patients in 13 European countries between 2008 and 2024 (Figure S1).

Nine proton‐pump inhibitor (PPI)‐based regimens were evaluated: clarithromycin–amoxicillin (Triple‐CA), clarithromycin–metronidazole (Triple‐CM), amoxicillin–levofloxacin (Triple‐AL), and amoxicillin–metronidazole (Triple‐AM) triple therapies; clarithromycin–amoxicillin–tinidazole/metronidazole sequential (Seq‐CAT/M) and concomitant (Conco‐CAT/M) therapies; clarithromycin–amoxicillin–bismuth quadruple therapy (Quad‐CAB); metronidazole–tetracycline–bismuth quadruple therapy (Quad‐MTcB); and single‐capsule bismuth quadruple therapy (Sc‐BQT). PPI types (omeprazole, lansoprazole, pantoprazole, rabeprazole, esomeprazole) and dosages were standardized as omeprazole‐equivalent potency according to intragastric pH > 4 duration over 24 h (pH 4‐time) and classified as low (4.5–27 mg), standard (32–40 mg), or high (54–128 mg), corresponding approximately to omeprazole 20, 40, or 80 mg twice daily, respectively [14, 15, 16]. Treatment durations of 7, 10, or 14 days were evaluated for all regimens. Compliance was assessed at follow‐up and considered adequate when ≥ 90% of prescribed drugs were taken.

### 2.3 Statistical Analysis

Continuous variables were summarized as means with standard deviations and categorical variables as frequencies and percentages. Group differences were assessed using chi‐square or Kruskal‐Wallis tests.

Three complementary approaches evaluated the relationship between antibiotic resistance and mITT outcomes:

First, Spearman's rank correlations (*ρ*) were calculated between regimen‐specific country‐year eradication rates and matched clarithromycin, metronidazole, and levofloxacin resistance estimates for each temporal window.

Second, candidate covariates were selected based on previous Hp‐EuReg analyses [17] and univariate logistic regressions were performed with mITT outcome as the dependent variable. Associated variables (*p* < 0.05) entered a backward stepwise multivariate model. Covariates included: age; sex (female [reference]); country: (Spain [reference], Belgium, Bulgaria, France, Germany, Israel, Italy, Poland, Portugal, Russia, Slovenia, the Netherlands, and the United Kingdom); prescription year (2013 [reference]–2024); regimen (Sc‐BQT [reference], Triple‐CA, Triple‐CM, Conco‐CAT/M, Seq‐CAT/M, Quad‐CAB, or Quad‐MTcB); PPI dosage (low [reference], standard, or high); duration (7 days [reference], 10, or 14 days); and compliance (< 90% [reference] vs. ≥ 90%). Resistance estimates were subsequently added to assess their contribution. Results were reported as Odds Ratios (ORs) with 95% confidence intervals (CIs). Collinearity was assessed using generalized variance inflation factors (VIF), with VIF ≥ 5 indicating collinearity (*performance* R‐package [18]), and discrimination was evaluated using the area under the curve (AUC), accuracy, sensitivity, and specificity (*pROC* R‐package [19]). Regimen‐specific analyses included only the corresponding antibiotic resistance and were restricted to therapies with adequate sample size. All models used 10,000 mITT‐stratified bootstrap replicates (*car, bayestestR, boot.pval* R‐packages [20, 21, 22]).

Third, three Random Forest models—one per resistance timeframe—were trained with mITT as the target and all remaining variables as predictors (*randomForest* R‐package [23]). Data were divided into training (80%) and test (20%) sets. Models used 1000 trees with class‐balanced bootstrap sampling and were evaluated by stratified 10‐fold cross‐validation using class‐specific metrics. Variable importance was estimated by permutation‐based mean decrease in accuracy, and partial dependence plots characterized the marginal effects of predictors on predicted mITT.

All analyses were conducted using R v4.5.0 [24].

## 3 Results

### 3.1 Study Population

From 41,310 cases extracted from Hp‐EuReg with available resistance data, 37,659 met the eligibility criteria for the mITT analysis (Failure *n* = 3839 [10%]; Success *n* = 33,820 [90%]) (Table 1) (Figure S1). The patients with available resistance data between 2013 and 2024 were recruited from 13 European countries. The largest contributors were Spain (*n* = 20,571 [50%]), Russia (*n* = 9367 [23%]), and Italy (*n* = 4229 [10%]), and the year with the highest recruitment was 2023 (*n* = 4200 [10%]). The mean participants' age was 51 ± 15 years, and 61% were female. Most patients were Caucasian (*n* = 35,527 [86%]) (Table 1).

**TABLE 1:** Baseline demographic, clinical, and treatment characteristics of the study population, overall and stratified by first‐line empirical treatment regimen.

Variable	*N*	Overall	Sc‐BQT	Triple‐CA	Triple‐CM	Triple‐AM	Triple‐AL	Conco‐CAT/M	Seq‐CAT/M	Quad‐CAB	Quad‐MTcB	*p* ^b^
		*N* = 41.310^a^	*N* = 9603^a^	*N* = 10.466^a^	*N* = 1099^a^	*N* = 284^a^	*N* = 435^a^	*N* = 8927^a^	*N* = 2980^a^	*N* = 6768^a^	*N* = 748^a^	
**Modified Intention‐to‐treat**	37,659											< 10^−3^
Failure		3839 (10.19%)	604 (6.60%)	1330 (14.73%)	211 (21.51%)	45 (22.39%)	73 (18.16%)	861 (10.13%)	240 (9.54%)	423 (6.82%)	52 (7.67%)	
Success		33,820 (89.81%)	8541 (93.40%)	7701 (85.27%)	770 (78.49%)	156 (77.61%)	329 (81.84%)	7640 (89.87%)	2275 (90.46%)	5782 (93.18%)	626 (92.33%)	
Missing		3651	458	1435	118	83	33	426	465	563	70	
**Country**	41,310											< 10^−3^
Spain		20,571 (49.80%)	7811 (81.34%)	2977 (28.44%)	148 (13.47%)	20 (7.04%)	145 (33.33%)	7841 (87.83%)	232 (7.79%)	1277 (18.87%)	120 (16.04%)	
Belgium		71 (0.17%)	1 (0.01%)	62 (0.59%)	1 (0.09%)	0 (0.00%)	0 (0.00%)	7 (0.08%)	0 (0.00%)	0 (0.00%)	0 (0.00%)	
Bulgaria		177 (0.43%)	0 (0.00%)	3 (0.03%)	1 (0.09%)	1 (0.35%)	3 (0.69%)	4 (0.04%)	156 (5.23%)	0 (0.00%)	9 (1.20%)	
France		183 (0.44%)	49 (0.51%)	5 (0.05%)	1 (0.09%)	0 (0.00%)	0 (0.00%)	3 (0.03%)	69 (2.32%)	1 (0.01%)	55 (7.35%)	
Germany		443 (1.07%)	183 (1.91%)	241 (2.30%)	7 (0.64%)	8 (2.82%)	1 (0.23%)	1 (0.01%)	0 (0.00%)	0 (0.00%)	2 (0.27%)	
Israel		298 (0.72%)	0 (0.00%)	47 (0.45%)	3 (0.27%)	6 (2.11%)	7 (1.61%)	210 (2.35%)	19 (0.64%)	1 (0.01%)	5 (0.67%)	
Italy		4229 (10.24%)	916 (9.54%)	227 (2.17%)	8 (0.73%)	6 (2.11%)	126 (28.97%)	500 (5.60%)	2398 (80.47%)	0 (0.00%)	48 (6.42%)	
Poland		625 (1.51%)	354 (3.69%)	2 (0.02%)	5 (0.45%)	118 (41.55%)	19 (4.37%)	2 (0.02%)	13 (0.44%)	0 (0.00%)	112 (14.97%)	
Portugal		762 (1.84%)	285 (2.97%)	156 (1.49%)	7 (0.64%)	6 (2.11%)	3 (0.69%)	246 (2.76%)	59 (1.98%)	0 (0.00%)	0 (0.00%)	
Russia		9367 (22.67%)	2 (0.02%)	3150 (30.10%)	37 (3.37%)	55 (19.37%)	109 (25.06%)	109 (1.22%)	23 (0.77%)	5487 (81.07%)	395 (52.81%)	
Slovenia		4026 (9.75%)	2 (0.02%)	3157 (30.16%)	835 (75.98%)	10 (3.52%)	6 (1.38%)	4 (0.04%)	10 (0.34%)	2 (0.03%)	0 (0.00%)	
The Netherlands		120 (0.29%)	0 (0.00%)	97 (0.93%)	4 (0.36%)	5 (1.76%)	14 (3.22%)	0 (0.00%)	0 (0.00%)	0 (0.00%)	0 (0.00%)	
United Kingdom		438 (1.06%)	0 (0.00%)	342 (3.27%)	42 (3.82%)	49 (17.25%)	2 (0.46%)	0 (0.00%)	1 (0.03%)	0 (0.00%)	2 (0.27%)	
**Year**	41,310											< 10^−3^
2013		2628 (6.36%)	5 (0.05%)	1165 (11.13%)	80 (7.28%)	49 (17.25%)	40 (9.20%)	727 (8.14%)	431 (14.46%)	68 (1.00%)	63 (8.42%)	
2014		3385 (8.19%)	7 (0.07%)	1622 (15.50%)	254 (23.11%)	50 (17.61%)	41 (9.43%)	851 (9.53%)	372 (12.48%)	115 (1.70%)	73 (9.76%)	
2015		3083 (7.46%)	20 (0.21%)	1132 (10.82%)	307 (27.93%)	21 (7.39%)	41 (9.43%)	1016 (11.38%)	286 (9.60%)	243 (3.59%)	17 (2.27%)	
2016		3792 (9.18%)	666 (6.94%)	981 (9.37%)	253 (23.02%)	11 (3.87%)	36 (8.28%)	926 (10.37%)	86 (2.89%)	825 (12.19%)	8 (1.07%)	
2017		3462 (8.38%)	918 (9.56%)	886 (8.47%)	44 (4.00%)	12 (4.23%)	18 (4.14%)	785 (8.79%)	307 (10.30%)	480 (7.09%)	12 (1.60%)	
2018		3577 (8.66%)	811 (8.45%)	901 (8.61%)	32 (2.91%)	5 (1.76%)	14 (3.22%)	399 (4.47%)	342 (11.48%)	1046 (15.46%)	27 (3.61%)	
2019		3265 (7.90%)	952 (9.91%)	994 (9.50%)	29 (2.64%)	16 (5.63%)	25 (5.75%)	433 (4.85%)	246 (8.26%)	515 (7.61%)	55 (7.35%)	
2020		2828 (6.85%)	975 (10.15%)	462 (4.41%)	21 (1.91%)	15 (5.28%)	43 (9.89%)	583 (6.53%)	177 (5.94%)	492 (7.27%)	60 (8.02%)	
2021		3977 (9.63%)	1283 (13.36%)	678 (6.48%)	27 (2.46%)	27 (9.51%)	57 (13.10%)	837 (9.38%)	241 (8.09%)	756 (11.17%)	71 (9.49%)	
2022		4100 (9.92%)	1534 (15.97%)	593 (5.67%)	20 (1.82%)	31 (10.92%)	25 (5.75%)	830 (9.30%)	245 (8.22%)	676 (9.99%)	146 (19.52%)	
2023		4200 (10.17%)	1450 (15.10%)	571 (5.46%)	20 (1.82%)	23 (8.10%)	45 (10.34%)	927 (10.38%)	151 (5.07%)	891 (13.16%)	122 (16.31%)	
2024		3013 (7.29%)	982 (10.23%)	481 (4.60%)	12 (1.09%)	24 (8.45%)	50 (11.49%)	613 (6.87%)	96 (3.22%)	661 (9.77%)	94 (12.57%)	
**Sex**	41,276											< 10^−3^
Female		25,106 (60.82%)	5857 (61.02%)	6162 (58.88%)	712 (64.79%)	154 (54.23%)	287 (65.98%)	5421 (60.82%)	1766 (59.34%)	4243 (62.80%)	504 (67.38%)	
Male		16,170 (39.18%)	3742 (38.98%)	4304 (41.12%)	387 (35.21%)	130 (45.77%)	148 (34.02%)	3492 (39.18%)	1210 (40.66%)	2513 (37.20%)	244 (32.62%)	
Missing		34	4	0	0	0	0	14	4	12	0	
**Age**	41,310											< 10^−3^
Mean (SD)		50.60 (15.02)	51.49 (15.05)	50.28 (15.12)	53.17 (15.01)	49.62 (16.54)	51.61 (14.32)	50.70 (15.06)	50.60 (15.02)	51.49 (15.05)	50.28 (15.12)	
**Ethnic background**	41,247											
Caucasian		35,527 (86.13%)	8998 (93.75%)	9006 (86.33%)	1054 (95.91%)	210 (73.94%)	333 (76.73%)	8050 (90.32%)	2560 (85.99%)	4673 (69.08%)	643 (86.31%)	
Black		294 (0.71%)	75 (0.78%)	40 (0.38%)	1 (0.09%)	1 (0.35%)	6 (1.38%)	68 (0.76%)	81 (2.72%)	18 (0.27%)	4 (0.54%)	
Asian		564 (1.37%)	70 (0.73%)	113 (1.08%)	1 (0.09%)	5 (1.76%)	6 (1.38%)	53 (0.59%)	68 (2.28%)	243 (3.59%)	5 (0.67%)	
Other		4009 (9.72%)	364 (3.79%)	1073 (10.29%)	17 (1.55%)	25 (8.80%)	83 (19.12%)	465 (5.22%)	91 (3.06%)	1802 (26.64%)	89 (11.95%)	
Unknown/Not available		853 (2.07%)	91 (0.95%)	200 (1.92%)	26 (2.37%)	43 (15.14%)	6 (1.38%)	277 (3.11%)	177 (5.95%)	29 (0.43%)	4 (0.54%)	
Missing		63	5	34	0	0	1	14	3	3	3	
**Compliance**	40,001											< 10^−3^
No (< 90% drug intake)		1143 (2.86%)	245 (2.61%)	218 (2.20%)	7 (0.70%)	3 (1.32%)	13 (3.04%)	291 (3.33%)	240 (8.15%)	112 (1.69%)	14 (1.97%)	
Yes (≥ 90% drug intake)		38,858 (97.14%)	9145 (97.39%)	9691 (97.80%)	993 (99.30%)	225 (98.68%)	414 (96.96%)	8457 (96.67%)	2706 (91.85%)	6531 (98.31%)	696 (98.03%)	
Missing		1309	213	557	99	56	8	179	34	125	38	
**PPI dose**	41,310											< 10^−3^
Low		16,637 (40.27%)	4597 (47.87%)	4232 (40.44%)	640 (58.23%)	194 (68.31%)	202 (46.44%)	4047 (45.33%)	1597 (53.59%)	863 (12.75%)	265 (35.43%)	
Standard		11,993 (29.03%)	2418 (25.18%)	3044 (29.08%)	68 (6.19%)	50 (17.61%)	142 (32.64%)	2017 (22.59%)	87 (2.92%)	3852 (56.91%)	315 (42.11%)	
High		12,680 (30.69%)	2588 (26.95%)	3190 (30.48%)	391 (35.58%)	40 (14.08%)	91 (20.92%)	2863 (32.07%)	1296 (43.49%)	2053 (30.33%)	168 (22.46%)	
**Length (days)**	41,310											< 10^−3^
7		2784 (6.74%)	6 (0.06%)	1806 (17.26%)	792 (72.07%)	79 (27.82%)	17 (3.91%)	22 (0.25%)	0 (0.00%)	51 (0.75%)	11 (1.47%)	
10		20,458 (49.52%)	9456 (98.47%)	3611 (34.50%)	137 (12.47%)	94 (33.10%)	292 (67.13%)	2871 (32.16%)	2807 (94.19%)	890 (13.15%)	300 (40.11%)	
14		18,068 (43.74%)	141 (1.47%)	5049 (48.24%)	170 (15.47%)	111 (39.08%)	126 (28.97%)	6034 (67.59%)	173 (5.81%)	5827 (86.10%)	437 (58.42%)	
**Adverse event**	40,194											< 10^−3^
No		31,708 (78.89%)	7505 (79.87%)	8672 (86.90%)	822 (81.31%)	238 (86.23%)	357 (82.83%)	6563 (75.08%)	2504 (84.17%)	4506 (67.73%)	541 (73.91%)	
Yes		8486 (21.11%)	1891 (20.13%)	1307 (13.10%)	189 (18.69%)	38 (13.77%)	74 (17.17%)	2178 (24.92%)	471 (15.83%)	2147 (32.27%)	191 (26.09%)	
Missing		1116	207	487	88	8	4	186	5	115	16	
**Serious adverse event**	8410											0.05
No		8378 (99.62%)	1881 (99.42%)	1236 (99.60%)	185 (100.00%)	35 (100.00%)	71 (98.61%)	2110 (99.39%)	540 (100.00%)	2139 (99.91%)	181 (100.00%)	
Yes		32 (0.38%)	11 (0.58%)	5 (0.40%)	0 (0.00%)	0 (0.00%)	1 (1.39%)	13 (0.61%)	0 (0.00%)	2 (0.09%)	0 (0.00%)	
Missing		32,900	7711	9225	914	249	363	6804	2440	4627	567	
**Treatment indication**	37,352											< 10^−3^
Dyspepsia and other		31,103 (83.27%)	7452 (87.34%)	7416 (78.10%)	883 (83.38%)	214 (81.37%)	300 (77.52%)	6769 (84.84%)	2787 (95.77%)	4709 (78.04%)	573 (82.80%)	
Ulcer		6249 (16.73%)	1080 (12.66%)	2080 (21.90%)	176 (16.62%)	49 (18.63%)	87 (22.48%)	1210 (15.16%)	123 (4.23%)	1325 (21.96%)	119 (17.20%)	
Missing		3958	1071	970	40	21	48	948	70	734	56	
**Resistance to clarithromycin (current year)**	26,418											< 10^−3^
Mean (SD)		18.42 (10.16)	18.26 (7.94)	15.95 (9.84)	14.28 (5.70)	25.08 (12.64)	20.36 (10.24)	17.99 (7.13)	33.47 (14.62)	12.80 (4.46)	18.57 (7.28)	
Missing		14,892	3083	4441	162	138	148	1796	546	4034	544	
**Resistance to clarithromycin (previous year)**	28,797											< 10^−3^
Mean (SD)		18.34 (10.47)	18.87 (8.34)	15.42 (10.25)	15.40 (6.64)	29.73 (15.61)	22.44 (11.84)	18.28 (8.76)	31.96 (13.36)	11.81 (4.95)	15.54 (7.47)	
Missing		12,513	2199	4114	188	140	140	1212	352	3674	494	
**Resistance to clarithromycin (last 5 years)**	37,408											< 10^−3^
Mean, (SD)		17.62 (10.47)	19.65 (9.76)	13.96 (9.16)	14.42 (6.09)	25.96 (16.36)	23.47 (12.50)	19.46 (10.16)	30.83 (8.66)	9.93 (4.24)	14.43 (7.13)	
Missing		3902	433	1478	51	62	77	69	1	1514	217	
**Resistance to metronidazole (current year)**	24,960											< 10^−3^
Mean, (SD)		31.42 (10.14)	27.23 (6.37)	34.32,(10.95)	28.56 (9.39)	55.17 (22.61)	28.70 (8.45)	29.23 (6.17)	34.44 (10.72)	37.83 (14.18)	36.48 (12.19)	
Missing		16,350	3130	5244	678	142	148	1889	559	4010	550	
**Resistance to metronidazole (previous year)**	28,166											< 10^−3^
Mean, (SD)		31.16 (9.23)	27.67 (6.28)	34.85 (10.81)	34.94 (11.43)	53.41 (17.23)	31.23 (8.61)	30.07 (6.79)	32.51 (10.06)	33.21 (11.05)	35.90 (13.46)	
Missing		13,144	1748	4762	865	144	139	945	356	3666	519	
**Resistance to metronidazole (last 5 years)**	35,817											< 10^−3^
Mean, (SD)		31.80 (8.20)	30.49 (5.53)	31.38 (10.10)	29.83 (10.48)	48.11 (16.82)	32.36 (7.06)	31.87 (5.70)	33.14 (6.11)	32.81 (10.69)	36.88 (12.22)	
Missing		5493	553	2399	567	75	81	74	11	1514	219	
**Resistance to levofloxacin (current year)**	25,990											< 10^−3^
Mean, (SD)		18.65 (9.25)	20.44 (6.02)	15.32 (9.65)	3.86 (8.00)	11.75 (7.64)	20.92 (8.40)	19.38 (4.66)	30.99 (12.45)	15.11 (7.64)	18.09 (7.54)	
Missing		15,320	3169	4464	164	138	190	1856	761	4034	544	
**Resistance to levofloxacin (previous year)**	28,435											< 10^−3^
Mean, (SD)		18.91 (8.67)	20.55 (6.01)	15.34 (9.48)	4.06 (8.35)	10.74 (7.99)	22.07 (9.45)	19.26 (4.75)	29.31 (11.62)	18.05 (6.20)	17.15 (7.79)	
Missing		12,875	2255	4123	188	141	148	1261	591	3674	494	
**Resistance to levofloxacin (last 5 years)**	33,846											< 10^−3^
Mean, (SD)		18.76 (9.05)	20.94 (6.79)	14.40 (9.72)	4.04 (8.17)	13.01 (9.94)	24.25 (9.72)	20.06 (6.07)	29.87 (7.34)	18.03 (5.81)	16.55 (8.14)	
Missing		7464	2519	1553	57	64	88	1337	1	1578	267	

*Note:* Continuous variables are presented as mean (standard deviation, SD) and categorical variables as *n* (%). *p* Values correspond to Pearson's Chi‐squared test for categorical variables and Kruskal‐Wallis rank sum test for continuous variables, comparing distributions across treatment groups. PPI dose was categorized as low (4.5–27 mg omeprazole equivalents twice daily; approximately omeprazole 20 mg twice daily), standard (32–40 mg; approximately omeprazole 40 mg twice daily), or high (54–128 mg; approximately omeprazole 80 mg twice daily). Adequate compliance was defined as ≥ 90% drug intake and inadequate compliance as < 90%.

Abbreviations: Conco‐CAT/M, concomitant clarithromycin‐amoxicillin‐triple therapy; PPI, proton‐pump inhibitor; Quad‐CAB, quadruple clarithromycin‐amoxicillin‐bismuth therapy; Quad‐MTcB, quadruple metronidazole‐tetracycline‐bismuth therapy; Sc‐BQT, single‐capsule bismuth quadruple therapy; SD, Standard deviation; Seq‐CAT/M, sequential clarithromycin‐amoxicillin‐metronidazole therapy; Triple‐AL, amoxicillin‐levofloxacin triple therapy; Triple‐AM, amoxicillin‐metronidazole triple therapy; Triple‐CA, clarithromycin‐amoxicillin triple therapy; Triple‐CM, clarithromycin‐metronidazole triple therapy.

^a^ *n* (%).

^b^ Pearson's Chi‐squared test; Kruskal‐Wallis rank sum test.

The most common first‐line empirical therapies were Triple‐CA (*n* = 10,466 [25%]), Sc‐BQT (*n* = 9603 [23%]), and Conco‐CAT/M (*n* = 8927 [22%]), followed by Quad‐CAB (*n* = 6768 [16%]) and Seq‐CAT/M (*n* = 2980 [7.2%]). Less frequent regimens included Triple‐CM (*n* = 1099 [2.7%]), Quad‐MTcB (*n* = 748 [1.8%]), Triple‐AL (*n* = 435 [1.0%]), and Triple‐AM (*n* = 284 [0.7%]) (Table 1).

The mean antibiotic resistance prevalence in the treatment year was 18% ± 10% for clarithromycin, 31% ± 10% for metronidazole, and 19% ± 9.3% for levofloxacin, with similar values for the prior‐year and five‐year average estimates (Table 1).

### 3.2 Correlation of Resistance Trends With Effectiveness

Overall, Spearman correlation analyses between resistance rates and eradication outcomes showed weak‐to‐moderate associations. The Triple‐AM therapy showed a significant negative correlation between prior‐year metronidazole resistance and treatment success (*ρ* = −0.52, *p* = 0.02) (Figure 1A). Quad‐MTcB showed a similar negative correlation with the five‐year mean metronidazole resistance (*ρ* = −0.35, *p* = 0.04) (Figure 1B). For Triple‐CM, significant negative correlations were observed with both five‐year mean clarithromycin resistance (*ρ* = −0.28, *p* = 0.04) (Figure 1C) and metronidazole resistance (*ρ* = −0.29, *p* = 0.04) (Figure 1D). No other regimens showed significant associations (Table S1).

**FIGURE 1:**
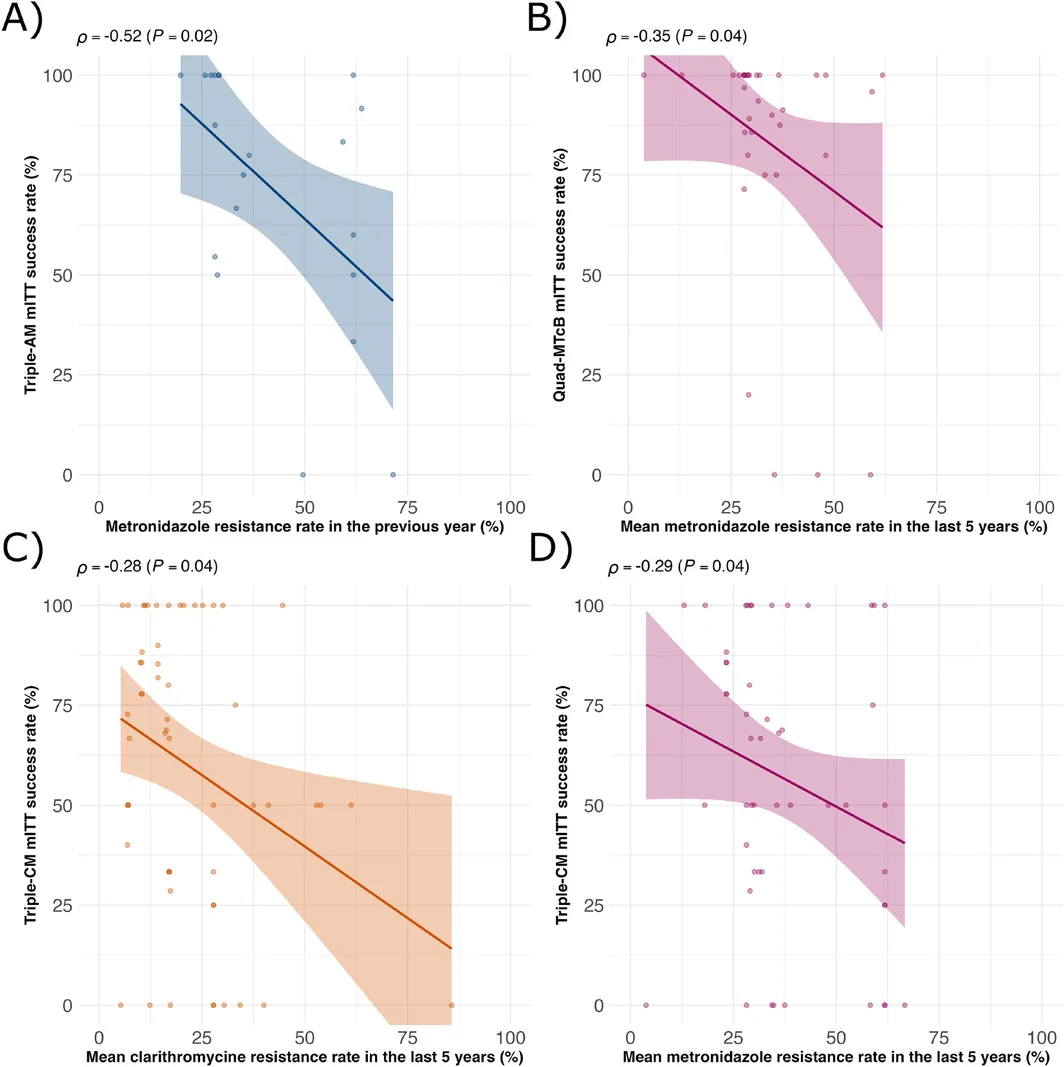
Correlation between eradication success in the modified intention‐to‐treat (mITT) population and resistance by treatment and country‐year. Panels show Spearman correlation coefficients (*ρ*) between eradication success and (A) previous‐year metronidazole resistance in patients receiving Triple‐AM therapy; (B) five‐year mean metronidazole resistance in patients receiving Quad‐MTcB therapy; and (C) five‐year mean clarithromycin and (D) metronidazole resistance in patients receiving Triple‐CM therapy. Each point represents a country‐year combination; lines show fitted linear trends with 95% confidence intervals. Quad‐MTcB, quadruple metronidazole–tetracycline–bismuth therapy; Triple‐AM, amoxicillin–metronidazole triple therapy; Triple‐CM, clarithromycin–metronidazole triple therapy.

### 3.3 Overall Associations Between Antibiotic Resistance and Effectiveness

Univariate and subsequent multivariate logistic regression models for the mITT population are shown in Table S2. In the model without antibiotic resistance variables, compliance, PPI dosage, and prescribed treatment remained significantly associated with treatment outcome (all *p* < 10^−3^) (Figure 2A). Adequate compliance and higher PPI dosage were associated with increased odds of treatment success, whereas the use of any regimen other than Sc‐BQT was associated with lower odds.

**FIGURE 2:**
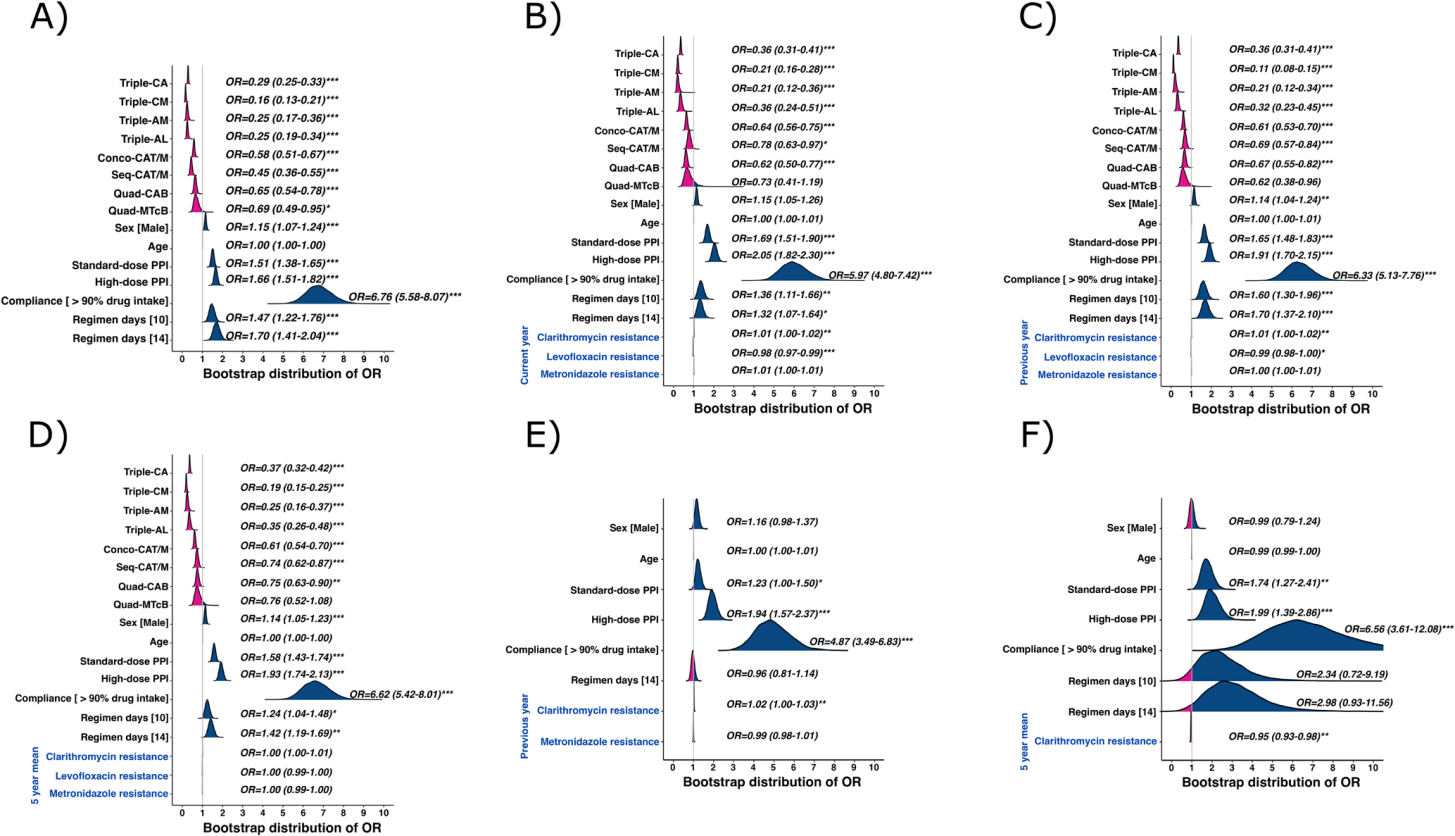
Bootstrapped multivariate logistic regression analyses for predictors of *H. pylori* eradication. Forest plots show bootstrapped odds ratio (OR) distributions from 10,000 replicates stratified by treatment outcome in the modified intention‐to‐treat (mITT) population. (A) Model without antibiotic resistance variables (*n* = 37,621; failures = 3835; successes = 33,786). Models including resistance rates for (B) the current year (*n* = 20,644; failures = 2413; successes = 18,231), (C) the previous year (*n* = 23,867; failures = 2630; successes = 21,237), and (D) the current and four preceding years (*n* = 29,492; failures = 3205; successes = 26,287). (E) Model restricted to concomitant therapy (Conco‐CAT/M), assessing previous‐year clarithromycin and metronidazole resistance (*n* = 6891; failures = 722; successes = 6169). (F) Model restricted to quadruple clarithromycin–amoxicillin–bismuth therapy (Quad‐CAB), assessing current‐year and five‐year mean clarithromycin resistance (*n* = 4827; failures = 349; successes = 4478). All models were adjusted for sex, age, proton pump inhibitor (PPI) potency, treatment duration, and compliance. Panels A–B were additionally adjusted for country and year (not shown). Panels B–F were additionally adjusted for the corresponding clarithromycin, levofloxacin, and metronidazole resistance rates. Reference categories were single‐capsule bismuth quadruple therapy (Sc‐BQT), female sex, low‐dose PPI potency, 7‐day treatment, and inadequate compliance. PPI dose was categorized as low (4.5–27 mg omeprazole equivalents twice daily; approximately omeprazole 20 mg twice daily), standard (32–40 mg; approximately omeprazole 40 mg twice daily), or high (54–128 mg; approximately omeprazole 80 mg twice daily). Adequate compliance was defined as ≥ 90% drug intake and inadequate compliance as < 90%. *p < 0.05, **p < 0.01, ***p < 0.001. Conco‐CAT/M, concomitant clarithromycin–amoxicillin–metronidazole therapy; OR, odds ratio; PPI, proton pump inhibitor; Quad‐CAB, quadruple clarithromycin–amoxicillin–bismuth therapy; Quad‐MTcB, quadruple metronidazole–tetracycline–bismuth therapy; Sc‐BQT, single‐capsule bismuth quadruple therapy; Seq‐CAT/M, sequential clarithromycin–amoxicillin–metronidazole therapy; Triple‐AL, amoxicillin–levofloxacin triple therapy; Triple‐AM, amoxicillin–metronidazole triple therapy; Triple‐CA, clarithromycin–amoxicillin triple therapy; Triple‐CM, clarithromycin–metronidazole triple therapy.

Multivariate models incorporating resistance rates for clarithromycin, metronidazole, and levofloxacin (current year values) are shown in Table S3. In this case, neither clarithromycin, levofloxacin, nor metronidazole resistance was significantly associated with treatment success (clarithromycin: *p* = 0.79; levofloxacin: *p* = 0.27; metronidazole: *p* = 0.23).

The VIF values for country (1146), year (16), resistance to clarithromycin (67), resistance to levofloxacin (18), and resistance to metronidazole (15) demonstrated substantial collinearity (Table S4), as resistance estimates were defined at the country‐year level. Consequently, *Country* and *Year* were excluded from the final multivariate model [25]. Discrimination was modest across models (AUC≈0.67–0.68) (Table S5), and inclusion of resistance variables did not improve AUC compared with the initial model.

The final model included the treatment prescribed, sex, age, PPI dosage, treatment duration, compliance, and resistance variables (Figure 2B). In this case, clarithromycin resistance showed a minimal increase in success odds (Bootstrap OR 1.01 [95% CI 1.00–1.02]; *p* = 3 × 10^−3^), metronidazole resistance a small decrease (Bootstrap OR 0.98 [95% CI 0.97–0.99]; *p* < 10^−3^), and levofloxacin resistance no meaningful association (*p* = 0.07).

When prior‐year resistance rates were included in the model instead (Figure 2C), clarithromycin resistance showed a positive association with treatment success (Bootstrap OR 1.01 [95% CI 1.00–1.02]; *p* = 0.01); metronidazole showed a slight decrease (Bootstrap OR 0.99 [95% CI 0.98–1.00]; *p* = 0.03), and levofloxacin resistance was not associated with eradication (*p* = 0.11). Other covariate associations remained as in the model without resistances: higher PPI potency and good compliance showed the strongest positive effects, while several regimens showed lower eradication odds compared with Sc‐BQT.

In the five‐year average resistance model, none of the three resistance variables were significantly associated with treatment success (clarithromycin: *p* = 0.29; metronidazole: *p* = 0.25; levofloxacin: *p* = 0.39) (Figure 2D). As in previous models, PPI potency and compliance maintained a strong positive association, while regimens like Triple‐CM and Triple‐AM were consistently associated with lower eradication odds compared to Sc‐BQT.

For the regimen‐level analyses, in Conco‐CAT/M (Figure 2E), prior‐year clarithromycin resistance was positively associated with treatment success (Bootstrap OR 1.02 [95% CI 1.00–1.03]; *p* < 0.01), whereas prior‐year metronidazole resistance was negatively associated (Bootstrap OR 0.99 [95% CI 0.98–1.01]; *p* = 0.01). In Quad‐CAB (Figure 2F), five‐year average clarithromycin resistance was associated with reduced treatment success (Bootstrap OR 0.95 [95% CI 0.93–0.98]; *p* < 10^−3^). For other therapies, no resistance rate was associated with treatment success in any timeframe (Tables S6–, S8).

### 3.4 Variable Prioritization

Predictive performance was consistent across the three Random Forest classification models, with cross‐validation mean percent correctly classified ranging from 66% to 68% (Table S9).

Variable importance analyses (Table S10) showed compliance, PPI dosage, and regimen duration as consistently dominant predictors (Figure 3). Sc‐BQT also ranked among the top predictors, whereas other regimens varied by model. Resistance variables generally ranked lower and contributed less to model accuracy than treatment‐related factors.

**FIGURE 3:**
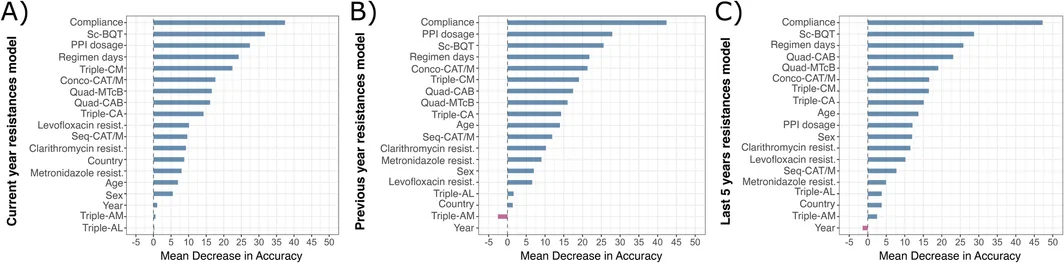
Variable importance from Random Forest models of eradication success in the modified intention‐to‐treat (mITT) population. Results are shown for the (A) current‐year, (B) previous‐year, and (C) five‐year mean resistance models. Bar plots show Mean Decrease in Accuracy, indicating each predictor's contribution to classification accuracy. Conco‐CAT/M, concomitant clarithromycin‐amoxicillin‐triple therapy; PPI, proton‐pump inhibitor; Quad‐CAB, quadruple clarithromycin‐amoxicillin‐bismuth therapy; Quad‐MTcB, quadruple metronidazole‐tetracycline‐bismuth therapy; Sc‐BQT, single‐capsule bismuth quadruple therapy; Seq‐CAT/M, sequential clarithromycin‐amoxicillin‐metronidazole therapy; Triple‐AL, amoxicillin‐levofloxacin triple therapy; Triple‐AM, amoxicillin‐metronidazole triple therapy; Triple‐CA, clarithromycin‐amoxicillin triple therapy; Triple‐CM, clarithromycin‐metronidazole triple therapy.

Partial dependence plots (Figure 4) showed that good compliance, higher PPI dose, and longer treatment duration were consistently associated with higher predicted eradication probabilities. Sc‐BQT treatment also had a positive marginal effect, whereas resistance rates showed weak or inconsistent patterns.

**FIGURE 4:**
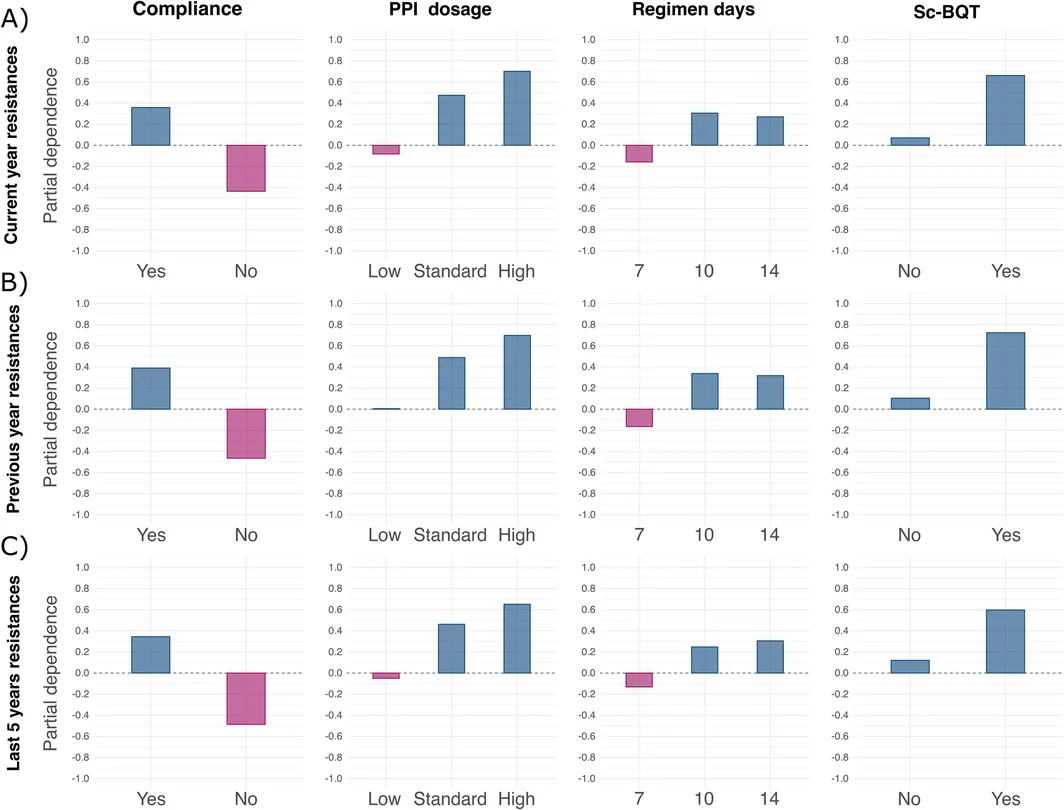
Partial dependence of treatment‐related variables on predicted treatment success. Plots show compliance, PPI dose, treatment duration, and Sc‐BQT treatment across (A) current‐year, (B) previous‐year, and (C) five‐year mean resistance models. Y‐axes show the average predicted probability of treatment success (mITT = 1) as each variable changes while all others remain constant. PPI dose was categorized as low (4.5–27 mg omeprazole equivalents twice daily; approximately omeprazole 20 mg twice daily), standard (32–40 mg; approximately omeprazole 40 mg twice daily), or high (54–128 mg; approximately omeprazole 80 mg twice daily). Adequate compliance was defined as ≥ 90% drug intake and inadequate compliance as < 90%. PPI, proton‐pump inhibitor; Sc‐BQT, single‐capsule bismuth quadruple therapy.

## 4 Discussion

This study evaluated whether literature‐derived *H. pylori* antibiotic resistance rates can reliably predict real‐world eradication outcomes among patients receiving first‐line empirical therapy. Although antimicrobial resistance is a well‐established driver of treatment failure, our results indicate that published resistance estimates are poor predictors of individual therapy success and may not adequately capture the factors influencing clinical outcome.

Across the three predefined resistance timeframes—same‐year, prior‐year, and five‐year average resistance; representing contemporaneous, recently available, and medium‐term background resistance—the impact on eradication success was limited and inconsistent. Scatterplots showed wide dispersion around regression lines, indicating that most variability remained unexplained. Logistic regression analyses revealed non‐significant and occasionally counterintuitive associations between clarithromycin, metronidazole, and levofloxacin resistance and eradication success, with generally small effect sizes, which should not be interpreted as protective biological effects, but rather as consequences of confounding. Similarly, random forest models prioritized compliance, PPI dosage, treatment duration, and specific regimens over antibiotic resistance.

The lack of concordance between aggregated resistance data and eradication rates may stem from several methodological and biological factors. A key limitation is the mismatch in scale: literature‐based resistance rates reflect population‐level estimates, whereas Hp‐EuReg measures success at the treatment‐case level. Although matched to country‐year pairs, *H. pylori* resistance varies between and within countries [26], meaning that broad aggregated estimates may not accurately reflect the resistance environment of the patients. At the individual level, resistance is a microbiological feature of the infecting strain; at the epidemiological level, it only reflects the average probability that a patient from a given country and year harbors a resistant strain. It cannot identify which patients carry resistant bacteria or capture mixed infections, heteroresistance, bacterial load, virulence factors, or adherence‐related failure. Thus, even valid population‐level estimates may be too imprecise to predict patient‐level eradication, likely explaining their limited predictive performance.

The quality and comparability of resistance data across studies constitute an additional source of uncertainty, as reporting is frequently incomplete. Documenting resistance to a single antibiotic does not guarantee the systematic evaluation of others, thereby introducing ascertainment bias when unmeasured co‐resistance is classified as isolated. This misclassification overlooks the significant implications of multidrug resistance for eradication efforts.

Published studies frequently derive from selected populations—including tertiary‐care referrals, patients with prior treatment failure, and endoscopy cohorts—whose baseline risk and clinical profile differ from those of typical first‐line patients. Small or geographically restricted samples are also extrapolated to entire country‐year strata, introducing measurement error, particularly in settings with substantial regional variation in antibiotic use, prescribing practices, and healthcare access. Publication delays mean that even year‐specific estimates may not be available at prescribing; although prior‐year resistance was examined to mitigate this limitation, it cannot fully account for reporting lags, missing data, or evolving treatment patterns. These limitations are consistent with previous concerns that reliable local resistance and eradication data are often difficult to generate, as susceptibility testing is not routinely performed and post‐treatment confirmation is inconsistent [27].

Our findings underscore the impact of modifiable, resistance independent factors on eradication outcomes. As in prior analyses from the Hp‐EuReg [17, 28], good compliance, higher PPI dose (at least 40 mg omeprazole equivalent twice daily), and longer treatment duration (usually 14 days) strongly predicted success. Sc‐BQT comprising metronidazole, tetracycline, and bismuth maintained > 90% effectiveness even in settings with higher reported resistance. Previous literature supports bismuth quadruple therapy as a key strategy to overcome declining clarithromycin‐based triple‐therapy efficacy in the context of rising resistance [29]. More recent evidence comparing susceptibility‐guided and empirical first‐line therapy suggests that its incremental benefit is attenuated (or even canceled) when bismuth quadruple therapy is the empirical comparator, supporting the latter as a high‐performing option when individual susceptibility data are unavailable [30]. This regimen‐dependent effect can help explain the limited predictive value observed for metronidazole resistance. Despite being common, metronidazole resistance has been shown to have a relatively limited impact on treatment success and can be partly overcome by higher doses, longer treatment durations, and the addition of bismuth to the regimen [29]. Therefore, if metronidazole resistance does not translate directly into failure of metronidazole‐containing therapies, the limited predictive value of population‐level metronidazole resistance observed in our study is not unexpected. Furthermore, this might help explain why modifiable treatment‐related factors, such as dose, duration, and regimen selection, emerged as more relevant predictors in our analyses.

The overall prevalence of resistance may also have influenced the associations detectable at the population level. Mean clarithromycin and levofloxacin resistance in the included observations was moderate, at approximately 18%–19%—just above the 15% resistance threshold used in clinical guidelines—although substantial variability was observed across country‐year estimates. However, the 15% threshold is primarily intended to guide empirical treatment selection and should not be interpreted as a biological cut‐off for the effect of resistance on treatment success. Therefore, the weak association in our analyses is more likely reflecting the limited ability of aggregated resistance data to reflect individual susceptibility and eradication likelihood, rather than an absence of a clinically relevant effect of resistance itself.

Another possible explanation for the counterintuitive associations could be that, in settings where resistance is unknown or suspected to be high, clinicians may employ more effective regimens [30], thereby mitigating the impact of resistance prevalence on observed eradication outcomes. Thus, a weak population‐level association between resistance estimates and treatment success may indicate that available resistance data are already incorporated into clinical practice guidelines, ultimately highlighting their utility.

Taken together, these findings suggest that literature‐derived resistance estimates may not adequately capture the conditions under which treatment is delivered in routine clinical practice. This reinforces the need for continuous, standardized, clinically integrated resistance surveillance. Until such data are routinely available, treatment decisions should prioritize evidence‐based regimens, adequate acid suppression, adherence optimisation, locally demonstrated effectiveness, and often the use of bismuth‐containing quadruple therapy. This does not diminish the relevance of antimicrobial resistance; rather, clinically informative estimates must be local, timely, standardized, and explicitly linked to treatment outcomes, serving as contextual indicators rather than direct predictors of individual eradication success.

The study has some limitations. The analysis specifically evaluated resistance to clarithromycin, levofloxacin, and metronidazole; therefore, the association between resistance to other antibiotics—such as amoxicillin and tetracycline—and treatment effectiveness remains to be assessed. These antibiotics were not included as resistance to amoxicillin and tetracycline is generally less frequent, as reported in previous studies [5, 31], and is therefore expected to have a lower population‐level impact than resistance to the other three. Accordingly, our conclusions regarding the limited predictive value of resistance data are restricted to the antibiotics evaluated. As discussed above, resistance exposure was assigned using aggregated literature‐derived estimates rather than individual susceptibility testing and therefore could not account for within‐country variation, patient‐level resistance status, mixed infections, or heteroresistance. In addition, published resistance data were heterogeneous in design, population selection, testing methods, reporting completeness, and timeliness, which may have introduced measurement error and limited comparability across country‐year strata. Even when adjusting for relevant variables, residual confounding could not be excluded in this real‐world observational setting.

Despite these limitations, the study has several notable strengths. It uses a large real‐world cohort, enabling assessment of first‐line empirical therapy under routine clinical conditions. Its multicountry, multiyear design provides broad geographic and temporal coverage, while evaluating multiple resistance timeframes allows testing associations across different clinical scenarios. Finally, the use of complementary analytical approaches strengthens the robustness of the findings, which highlight the important gap between population‐level resistance estimates and the determinants of real‐world *H. pylori* eradication success.

In conclusion, literature‐based antibiotic resistance data do not reliably predict real‐world first‐line empirical therapy success for *H. pylori* . In an era of rising antibiotic resistance, treatment success seems to depend more on appropriate regimen selection, patient adherence, treatment duration, PPI potency, and continuous monitoring of local clinical outcomes.

## Author Contributions

Ismail Kirrout and Nuria Montes summarized, interpreted and analyzed the data, performed all the statistical analysis, wrote the various drafts of the manuscript and approved the final submitted manuscript. Ismail Kirrout contributed to the interpretation and synthesis of the data, performed the statistical analyses, interpreted the results, prepared the figures and tables, drafted the manuscript and subsequent versions, critically revised them, and approved the final submission. Nuria Montes conceived and designed the analytical strategy, coordinated its development, performed the statistical analyses, reviewed and interpreted the results, drafted and critically revised each version of the manuscript, and approved the final submission. Olga P. Nyssen and Javier P. Gisbert planned and coordinated the study; summarized and interpreted the data, reviewed the drafts and are guarantors of the study. Olga P. Nyssen, Hp‐EuReg and WorldHpReg Scientific Director, performed the data extraction, supervised the monitoring and the quality checks, performed the interpretation, and synthesis, critically reviewed the various drafts of the manuscript, and approved the final submission. Javier P. Gisbert, Principal investigator of the registry, directed the project, obtained funding, designed the protocol, recruited patients, assisted with the data analysis, critically reviewed the various drafts of the manuscript, and approved the final submission. Ángeles Pérez‐Aísa, Bojan Tepes, Irina N. Voynovan, Samuel J. Martínez‐Domínguez, Alfredo J. Lucendo, Ludmila G. Vologzanina, Sayar R. Abdulkhakov, Dmitry S. Bordin, Mónica Sánchez Alonso, Luis Bujanda, and Javier P. Gisbert collected data, critically reviewed the various drafts of the manuscript, and approved the final submission. Pablo Parra performed the monitoring and quality check of the data, reviewed and approved the final submitted manuscript. Leticia Moreira, Pablo Parra, Olga P. Nyssen, Francis Mégraud, Colm O'Morain, and Javier P. Gisbert, all as members of the Hp‐EuReg and WorldHpReg Scientific Committee, critically reviewed the various drafts of the manuscript and approved the final submission.

## Funding

This project was promoted and funded by the European Helicobacter and Microbiota Study Group (EHMSG) and received support from the Spanish Association of Gastroenterology (AEG) and the Centro de Investigación Biomédica en Red de Enfermedades Hepáticas y Digestivas (CIBEREHD).

Hp‐EuReg was co‐funded by the European Union programme HORIZON (grant agreement number 101095359) and supported by the UK Research and Innovation (grant agreement number 10058099). However, the views and opinions expressed are those of the author(s) only and do not necessarily reflect those of the European Union or the Health and Digitial Executive Agency (HaDEA). Neither the European Union nor the granting authority can be held responsible for them. Hp‐EuReg was co‐funded by the European Union programme EU4Health (grant agreement number 101101252). Hp‐EuReg was partially co‐funded by Mayoly, Diasorin, Juvisé, Biocodex, and Zambon; however, no clinical data were accessible to these companies, and they were not involved in any stage of the Hp‐EuReg study (design, data collection, or statistical analysis).

## Disclosure

Patient and Public Involvement: Patients and/or the public were not involved in the design, conduct, reporting, or dissemination plans of this research.

## Ethics Statement

Institutional review board statement: The Hp‐EuReg protocol was approved by the Ethics Committee Hospital Universitario de la Princesa (Madrid, SPAIN), which acted as a reference Institutional Review Board (20 December 2012) (Ethics approval code: Hp‐EuReg). This research was conducted according to the guidelines of the Declaration of Helsinki, classified by the Spanish Agency for Medicines and Medical Devices, and prospectively registered at Clinical Trials.gov under the code NCT02328131.

## Conflicts of Interest

J.P.G. has served as speaker, consultant, and advisory member for or has received research funding from: Mayoly, Diasorin, Juvisé, Biocodex, and Zambon. O.P.N. has served as a speaker or has received research funding from Mayoly, Diasorin, Juvisé, Biocodex, and Zambon. L.M. has served as a speaker for Juvisé. The other authors declare no conflicts of interest.

## Supporting information


**Figure S1:** Patient flow chart with reasons for exclusion. Only countries encompassing patients with available resistance estimates for the predefined timeframes were eligible for inclusion. Among these, only patients with a recorded first‐line treatment regimen and confirmed follow‐up were included in the modified intention‐to‐treat (mITT) population and subsequently included in the analysis.

**Table S1:** Spearman correlation coefficients between antibiotic resistance rates and eradication success on the modified intention‐to‐treat population across treatment regimens.

**Table S2:** Univariate and multivariate logistic regression analyses of predictors for eradication success in the modified intention‐to‐treat population.

**Table S3:** Univariate and multivariate logistic regression analyses of predictors for eradication success in the modified intention‐to‐treat population including resistance variables.

**Table S4:** Variance inflation factor values for covariates included in the multivariate logistic regression model with antibiotic resistance variables.

**Table S5:** Discriminatory performance of the general prediction models.

**Table S6:** Bootstrapped multivariate logistic regression models of predictors of eradication success population, stratified by treatment regimen.

**Table S7:** Bootstrapped multivariate logistic regression models of predictors of eradication success population, stratified by treatment regimen.

**Table S8:** Bootstrapped multivariate logistic regression models of predictors of eradication success population, stratified by treatment regimen.

**Table S9:** Performance metrics of Random Forest models classifying modified intention‐to‐treat outcomes.

**Table S10:** Variable importance scores for Random Forest models.

**Data S1:** Hp‐EuReg investigators.

## Data Availability

Raw data were generated at AEG‐REDCap. All data relevant to the study are included in the article or uploaded as Supporting Information. De‐identified raw data referring to the current study are available from the WorldHpReg and Hp‐EuReg Scientific director and the principal investigator of the project (OPN and JPG respectively) upon request. Individual participant data will not be shared. The Hp‐EuReg and the WorldHpReg intellectual property was registered on March 24, 2024, under the Spanish Intellectual Property Office (registration number M‐001958/2024; asiento registral 16/2024/3986). In January 2026, WorldHpReg was included in the EMA–ERDW Catalogue of real‐world data sources (EU PAS number: EUPAS1000000912), enabling its use within European regulatory and research frameworks.
