# Supplementary material for: Lack of Usefulness of Literature‐Based Helicobacter pylori Resistance Data for Predicting First‐Line Empirical Treatment Effectiveness in the European Registry on H. pylori Management (Hp‐EuReg)
Source: Helicobacter. 2026 Nov 5;31(6):e70169. doi: 10.1111/hel.70169 (PMC13735993; doi:10.1111/hel.70169)
Supplement: Supplementary file 1 [file HEL-31-e70169-s012.pdf]

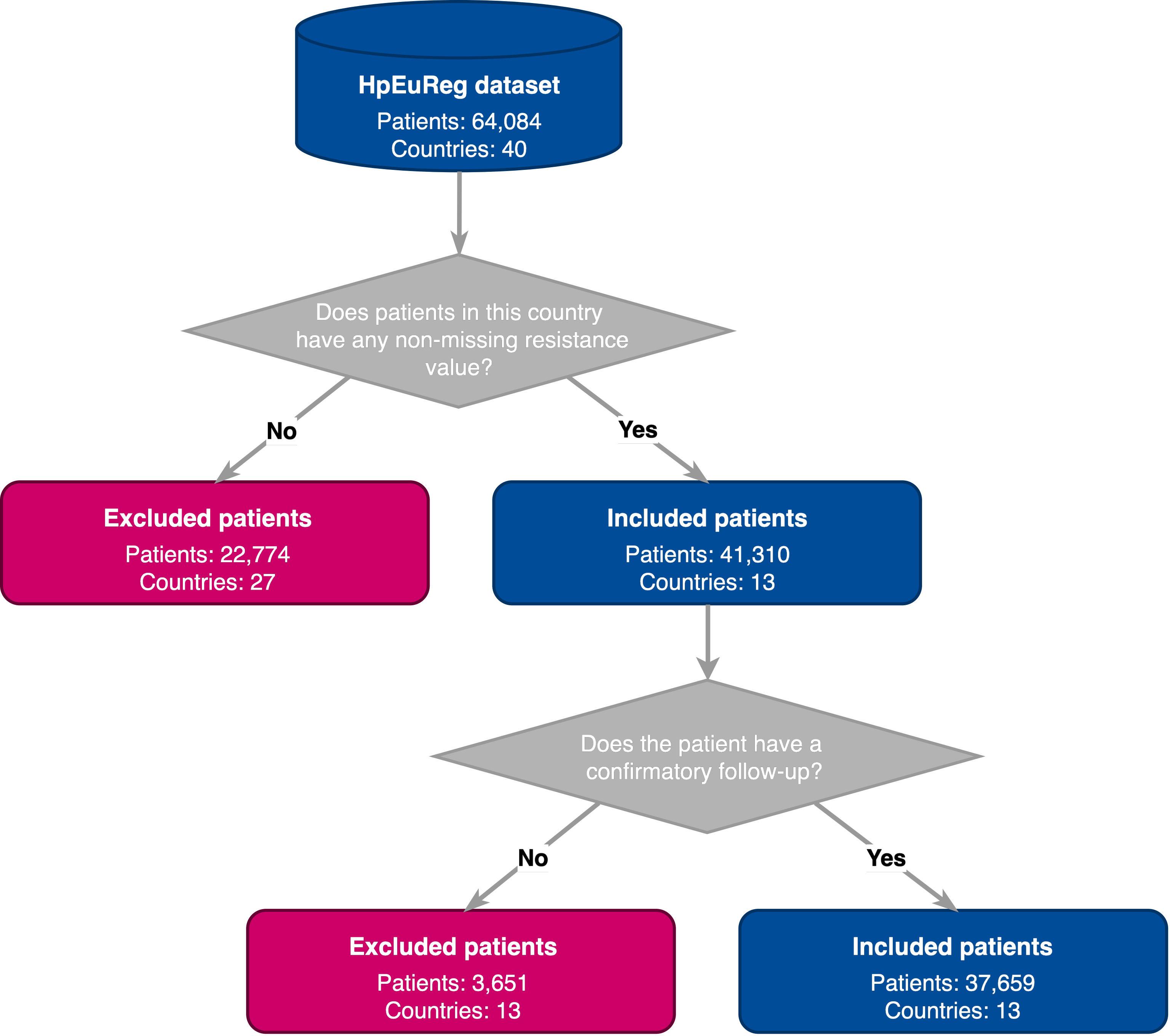
HpEuReg dataset
Patients: 64,084
Countries: 40
Does patients in this country have any non-missing resistance value?
No
Yes
Excluded patients
Patients: 22,774
Countries: 27
Included patients
Patients: 41,310
Countries: 13
Does the patient have a confirmatory follow-up?
No
Yes
Excluded patients
Patients: 3,651
Countries: 13
Included patients
Patients: 37,659
Countries: 13
