# Supplementary material for: Lack of Usefulness of Literature‐Based Helicobacter pylori Resistance Data for Predicting First‐Line Empirical Treatment Effectiveness in the European Registry on H. pylori Management (Hp‐EuReg)
Source: Helicobacter. 2026 Nov 5;31(6):e70169. doi: 10.1111/hel.70169 (PMC13735993; doi:10.1111/hel.70169)
Supplement: Supplementary file 2 [file HEL-31-e70169-s001.docx]

**Table S10.** Variable importance scores for Random Forest models.

| **Variable** | **Current year resistances model**  **MDA** | **Last year resistances model**  **MDA** | **Last 5 years resistance model**  **MDA** |
| --- | --- | --- | --- |
| **Sc-BQT** | 31.71 | 25.60 | 28.72 |
| **Triple-CA** | 14.24 | 14.27 | 15.14 |
| **Triple-CM** | 22.46 | 19.03 | 16.51 |
| **Triple-AM** | 0.58 | -2.55 | 2.52 |
| **Triple-AL** | 0.26 | 1.62 | 3.76 |
| **Conco-CAT/M** | 17.61 | 21.33 | 16.59 |
| **Seq-CAT/M** | 9.61 | 11.91 | 7.75 |
| **Quad-CAB** | 16.11 | 17.46 | 23.08 |
| **Quad-MTcB** | 16.61 | 15.99 | 19.06 |
| **Year** | 8.76 | 1.41 | 3.75 |
| **Country** | 1.04 | -7.05 | -1.40 |
| **Sex** | 5.50 | 7.02 | 12.00 |
| **Age** | 6.96 | 13.97 | 13.70 |
| **PPI dosage** | 27.43 | 27.91 | 12.10 |
| **Compliance** | 37.43 | 42.36 | 47.25 |
| **Regimen days** | 24.23 | 21.81 | 25.84 |
| **Resistance clarithromycin**  **(current year)** | 9.24 | - | - |
| **Resistance levofloxacin**  **(current year)** | 10.11 | - | - |
| **Resistance metronidazole**  **(current year)** | 7.99 | - | - |
| **Resistance clarithromycin**  **(previous year)** | - | 10.24 | - |
| **Resistance levofloxacin**  **(previous year)** | - | 6.59 | - |
| **Resistance metronidazole**  **(previous year)** | - | 9.06 | - |
| **Resistance clarithromycin**  **(current + 4 previous years)** | - | - | 11.51 |
| **Resistance levofloxacin**  **(current + 4 previous years)** | - | - | 10.17 |
| **Resistance metronidazole**  **(current + 4 previous years)** | - | - | 4.95 |

Mean decrease in accuracy (MDA) for all predictors in the current-year, previous-year, and last-five-years resistance models. Conco-CAT/M, concomitant clarithromycin-amoxicillin-triple therapy; MDA, mean decrease in accuracy; PPI, proton-pump inhibitor; Quad-CAB, quadruple clarithromycin-amoxicillin-bismuth therapy; Quad-MTcB, quadruple metronidazole-tetracycline-bismuth therapy; Sc-BQT, single-capsule bismuth quadruple therapy; Seq-CAT/M, sequential clarithromycin-amoxicillin-metronidazole therapy; Triple-AL, amoxicillin-levofloxacin triple therapy; Triple-AM, amoxicillin-metronidazole triple therapy; Triple-CA, clarithromycin-amoxicillin triple therapy; Triple-CM, clarithromycin-metronidazole triple therapy.
