# Supplementary material for: Lack of Usefulness of Literature‐Based Helicobacter pylori Resistance Data for Predicting First‐Line Empirical Treatment Effectiveness in the European Registry on H. pylori Management (Hp‐EuReg)
Source: Helicobacter. 2026 Nov 5;31(6):e70169. doi: 10.1111/hel.70169 (PMC13735993; doi:10.1111/hel.70169)
Supplement: Supplementary file 3 [file HEL-31-e70169-s002.docx]

**File S1.** Hp-EuReg investigators.

Fernando Bermejo, Department of Gastroenterology, Hospital Universitario de Fuenlabrada, Fuenlabrada, SPAIN Acquired data, critically reviewed the manuscript draft, and approved the submitted manuscript.

Natalia N. Dekhnich, Department of Faculty Therapy, Smolensk State Medical University, Smolensk, RUSSIA Acquired data, critically reviewed the manuscript draft, and approved the submitted manuscript.

Pilar Mata-Romero, Department of Gastroenterology, Hospital Universitario de Cáceres, Cáceres, SPAIN Acquired data, critically reviewed the manuscript draft, and approved the submitted manuscript.

Natalia Valerievna Bakulina, I.I. Mechnikov North-Western State Medical University of the Ministry of Health of the Russian Federation, Saint Petersburg, RUSSIA Acquired data, critically reviewed the manuscript draft, and approved the submitted manuscript.

Alla G. Kononova, Tver State Medical University, Ministry of Health of Russia, Tver, RUSSIA Acquired data, critically reviewed the manuscript draft, and approved the submitted manuscript.

Giuseppe Losurdo, Section of Gastroenterology, Department of Precision and Regenerative Medicine and Ionian Area, University of Bari, Bari, ITALY Acquired data, critically reviewed the manuscript draft, and approved the submitted manuscript.

Judith Gomez-Camarero, Department of Gastroenterology, Hospital Universitario de Burgos, Burgos, SPAIN Acquired data, critically reviewed the manuscript draft, and approved the submitted manuscript.

Alma Keco-Huerga, Department of Gastroenterology, Hospital Universitario Virgen de la Macarena, Sevilla, SPAIN Acquired data, critically reviewed the manuscript draft, and approved the submitted manuscript.

Pablo M. Wolfe García, Department of Gastroenterology and Hepatology, Hospital Sierrallana, Torrelavega, SPAIN Acquired data, critically reviewed the manuscript draft, and approved the submitted manuscript.

Jurij Bednarik, Department of Gastroenterology, Klinika Doktor 24 Ljubljana, SLOVENIA Acquired data, critically reviewed the manuscript draft, and approved the submitted manuscript.

Eduardo Iyo, Department of Gastroenterology, Hospital Universitari Son Espases, Palma (Mallorca), SPAIN Acquired data, critically reviewed the manuscript draft, and approved the submitted manuscript.

Antonia Perelló, Department of Gastroenterology, Hospital Universitari Son Espases, Palma (Mallorca), SPAIN Acquired data, critically reviewed the manuscript draft, and approved the submitted manuscript.

Luis Fernández-Salazar, Department of Gastroenterology, Hospital Clínico de Valladolid, Medicine Department, School of Medicine, Universidad de Valladolid, Valladolid, SPAIN Acquired data, critically reviewed the manuscript draft, and approved the submitted manuscript.

Noelia Alcaide, Department of Gastroenterology, Hospital Clínico de Valladolid, Valladolid, SPAIN Acquired data, critically reviewed the manuscript draft, and approved the submitted manuscript.

Benito Velayos, Department of Gastroenterology, Hospital Clínico de Valladolid, Valladolid, SPAIN Acquired data, critically reviewed the manuscript draft, and approved the submitted manuscript.

Pedro Almela, Department of Gastroenterology, Hospital General Universitario de Castellón, Ciencias de la Salud (Medicina), Universidad CEU Cardenal Herrera, Castellón, SPAIN Acquired data, critically reviewed the manuscript draft, and approved the submitted manuscript.

Ramón Pajares Villarroya, Gastroenterology Section, Hospital Universitario Infanta Sofía, Facultad de Medicina, Universidad Europea de Madrid, San Sebastián de los Reyes, SPAIN Acquired data, critically reviewed the manuscript draft, and approved the submitted manuscript.

Daniel Martin-Holgado, Department of Gastroenterology, Hospital Universitario de Cáceres, Cáceres, SPAIN Acquired data, critically reviewed the manuscript draft, and approved the submitted manuscript.

Miguel Fernández-Bermejo, Department of Gastroenterology, Hospital Parque San Francisco, Cáceres, SPAIN Acquired data, critically reviewed the manuscript draft, and approved the submitted manuscript.

Ian L. P. Beales, Norwich Medical School, University of East Anglia, Norwich, UNITED KINGDOM Acquired data, critically reviewed the manuscript draft, and approved the submitted manuscript.

Alisan Kahraman, Gastroenterology and Hepatology Department, Max Grundig Clinic, Bühl/Baden, GERMANY Acquired data, critically reviewed the manuscript draft, and approved the submitted manuscript.

Ana Beatriz Pozo Blanco, Department of Gastroenterology, Hospital Arnau Vilanova-Lliria, Valencia, SPAIN Acquired data, critically reviewed the manuscript draft, and approved the submitted manuscript.

Montserrat Planella, Department of Gastroenterology, Hospital Universitari Arnau de Vilanova, Institut de Recerca Biomèdica de Lleida (IRBLL), Lleida, SPAIN Acquired data, critically reviewed the manuscript draft, and approved the submitted manuscript.

Mikel Ganuza, Department of Gastroenterology, Hospital General de Granollers, Barcelona, SPAIN Acquired data, critically reviewed the manuscript draft, and approved the submitted manuscript.

Victor A. Kamburov, Department of Gastroenterology, BalkanMed Medical Center, Sofia, BULGARIA Acquired data, critically reviewed the manuscript draft, and approved the submitted manuscript.

Marina F. Osipenko, Novosibirsk State Medical University of the Ministry of Health of the Russian Federation, RUSSIA Acquired data, critically reviewed the manuscript draft, and approved the submitted manuscript.

Debora Compare, Department of Gastroenterology, Federico II University, Naples, ITALY Acquired data, critically reviewed the manuscript draft, and approved the submitted manuscript.

Georges Kamtoh, Hepatic Medical, Private Medical Center, Krakow, POLAND Acquired data, critically reviewed the manuscript draft, and approved the submitted manuscript.

Rosa Rosania, Department of Gastroenterology, Hepatology and Infectious Diseases, Otto von Guericke University Hospital, Magdeburg, GERMANY Acquired data, critically reviewed the manuscript draft, and approved the submitted manuscript.

Eva Barreiro Alonso, Department of Gastroenterology, Hospital Central de Asturias (HUCA), Department of Pharmacology, Instituto de Investigación Sanitaria del Principado de Asturias (ISPA), Oviedo, SPAIN Acquired data, critically reviewed the manuscript draft, and approved the submitted manuscript.

Maria A. Livzan, Omsk State Medical University of the Ministry of Health of the Russian Federation, RUSSIA Acquired data, critically reviewed the manuscript draft, and approved the submitted manuscript.

Teresa Angueira, Department of Gastroenterology, Hospital Universitario de Toledo, Toledo, SPAIN Acquired data, critically reviewed the manuscript draft, and approved the submitted manuscript.

Consuelo Ramírez, Department of Gastroenterology, Hospital Universitari Arnau de Vilanova, Institut de Recerca Biomèdica de Lleida (IRBLL), Lleida, SPAIN Acquired data, critically reviewed the manuscript draft, and approved the submitted manuscript.

Piotr Szredzki, Department of General Surgery, University of Rzeszów, Rzeszów, POLAND Acquired data, critically reviewed the manuscript draft, and approved the submitted manuscript.

Natalia Baryshnikova, Internal disease department of stomatology faculty, Pavlov First Saint Petersburg State Medical University, molecular microbiology department, Institute of Experimental Medicine, St-Petersburg, RUSSIA Acquired data, critically reviewed the manuscript draft, and approved the submitted manuscript.

Natalia Bakanova, Medical Center Mediceya, Izhevsk, RUSSIA Acquired data, critically reviewed the manuscript draft, and approved the submitted manuscript.

Pedro Delgado Guillena, Department of Gastroenterology, Hospital de Mérida, Mérida, SPAIN Acquired data, critically reviewed the manuscript draft, and approved the submitted manuscript.

Sheyla Montori Pina, Department of Gastroenterology, Hospital Universitario de Navarra (HUN), Navarrabiomed, Universidad Pública de Navarra (UPNA), IdiSNA, Pamplona, SPAIN Acquired data, critically reviewed the manuscript draft, and approved the submitted manuscript.

Eduardo Albéniz, Department of Gastroenterology, Hospital Universitario de Navarra (HUN), Navarrabiomed, Universidad Pública de Navarra (UPNA), IdiSNA, Pamplona, SPAIN Acquired data, critically reviewed the manuscript draft, and approved the submitted manuscript.

Piotr Eder, Department of Gastroenterology, Dietetics and Internal Medicine, Poznań University of Medical Sciences, Department of Gastroenterology, H. Święcicki University Hospital, Poznan, POLAND Acquired data, critically reviewed the manuscript draft, and approved the submitted manuscript.

Inna V. Savilova, I.I. Mechnikov North-Western State Medical University of the Ministry of Health of the Russian Federation RUSSIA Acquired data, critically reviewed the manuscript draft, and approved the submitted manuscript.

Henrique Fernandes-Mendes, Department of Gastroenterology, Centro Hospitalar Universitário de Santo António, ULS de Santo António, Porto, PORTUGAL Acquired data, critically reviewed the manuscript draft, and approved the submitted manuscript.

Miguel Suárez Matías, Department of Gastroenterology, Hospital Virgen de la Luz, Cuenca, SPAIN Acquired data, critically reviewed the manuscript draft, and approved the submitted manuscript.

María Badía Martínez, Department of Gastroenterology, Hopital General de la Defensa, Department of Gastroenterology, Hospital Universitario Miguel Servet, Zaragoza, SPAIN Acquired data, critically reviewed the manuscript draft, and approved the submitted manuscript.

Liya Nikolaevna Belousova, I.I. Mechnikov North-Western State Medical University of the Ministry of Health of the Russian Federation, Saint Petersburg, RUSSIA Acquired data, critically reviewed the manuscript draft, and approved the submitted manuscript.

Benito Hermida Pérez, Department of Gastroenterology, Hospital Valle del Nalón, Langreo, SPAIN Acquired data, critically reviewed the manuscript draft, and approved the submitted manuscript.

Ana Garre, Department of Gastroenterology, Hospital Universitario de La Princesa, Instituto de Investigación Sanitaria Princesa (IIS-Princesa), Universidad Autónoma de Madrid (UAM), and Centro de Investigación Biomédica en Red de Enfermedades Hepáticas y Digestivas (CIBERehd), Madrid, SPAIN Acquired data, critically reviewed the manuscript draft, and approved the submitted manuscript.

Natasa Brglez Jurecic, Interni Oddelek, Diagnostic Centre, Bled, SLOVENIA Acquired data, critically reviewed the manuscript draft, and approved the submitted manuscript.

Olga Kolokolnikova, Medsi Clinical Hospital, Moscow, RUSSIA Acquired data, critically reviewed the manuscript draft, and approved the submitted manuscript.

Paola Chaudarcas, Department of Gastroenterology, Hospital Universitario Infanta Sofía, San Sebastián de los Reyes, SPAIN Acquired data, critically reviewed the manuscript draft, and approved the submitted manuscript.

Luis Hernández, Department of Gastroenterology, Hospital Santos Reyes, Aranda de Duero, SPAIN Acquired data, critically reviewed the manuscript draft, and approved the submitted manuscript.

Regina I. Khlynova, Department of faculty therapy and geriatrics, Ural State Medical University, Ekaterinburg, RUSSIA Acquired data, critically reviewed the manuscript draft, and approved the submitted manuscript.

Antonio Cuadrado, Department of Gastroenterology and Hepatology, Hospital Universitario Marqués de Valdecilla, Clinical and Translational Research in Digestive Diseases, Valdecilla Research Institute (IDIVAL), Santander, SPAIN Acquired data, critically reviewed the manuscript draft, and approved the submitted manuscript.

Marta Pascual-Mato, Department of Gastroenterology and Hepatology, Clinical and Translational Research in Digestive Diseases, Valdecilla Research Institute (IDIVAL), Marqués de Valdecilla University Hospital, Santander, SPAIN Acquired data, critically reviewed the manuscript draft, and approved the submitted manuscript.

Ekaterina Y. Plotnikova, Department of Polyclinic Therapy, Postgraduate Training of Doctors and Nursing, Federal State Higher Education Institution Kemerovo State Medical University Ministry of Health of Russia, Kemerovo, RUSSIA Acquired data, critically reviewed the manuscript draft, and approved the submitted manuscript.

Mila Kovacheva-Slavova, Department of Gastroenterology, Queen Yoanna University Hospital, Medical University of Sofia, Sofia, BULGARIA Acquired data, critically reviewed the manuscript draft, and approved the submitted manuscript.

Ramiro Carreño Macián, Department of Gastroenterology, Hospital Quirónsalud Vitoria, Vitoria, SPAIN Acquired data, critically reviewed the manuscript draft, and approved the submitted manuscript.

Manon C.W. Spaander, Department of Gastroenterology and Hepatology, Erasmus University Medical Center, Rotterdam, THE NETHERLANDS Acquired data, critically reviewed the manuscript draft, and approved the submitted manuscript.

Guillem Soy, Department of Gastroenterology, Hospital Clínic de Barcelona, Institut Clínic de Malalties Digestives i Metabòliques (ICMDM), Barcelona, SPAIN Acquired data, critically reviewed the manuscript draft, and approved the submitted manuscript.

Pilar Pazo Mejide, Department of Gastroenterology, Hospital de Cruces, Barakaldo, SPAIN Acquired data, critically reviewed the manuscript draft, and approved the submitted manuscript.

Sergio Gil Rojas, Department of Gastroenterology, Hospital Virgen de la Luz, Cuenca, SPAIN Acquired data, critically reviewed the manuscript draft, and approved the submitted manuscript.

Carlos Maroto-Martín, Department of Gastroenterology, Hospital Río Hortega de Valladolid, Valladolid, SPAIN Acquired data, critically reviewed the manuscript draft, and approved the submitted manuscript.

Francisco J. Rancel-Medina, Department of Gastroenterology, Complejo Asistencial Universitario de Palencia, Palencia, SPAIN Acquired data, critically reviewed the manuscript draft, and approved the submitted manuscript.

Diego Burgos-Santamaría, Department of Gastroenterology and Hepatology, Hospital Universitario Ramón y Cajal, Madrid, SPAIN Acquired data, critically reviewed the manuscript draft, and approved the submitted manuscript.

Isabel Pérez-Martínez, Department of Gastroenterology, Hospital Universitario Central de Asturias, Instituto de Investigación Sanitaria del Principado de Asturias (ISPA), Oviedo, SPAIN Acquired data, critically reviewed the manuscript draft, and approved the submitted manuscript.

Giulia Fiorini, Cardiovascular Medicine Unit, Heart, Chest and Vascular Dept., IRCCS Azienda Ospedaliero-Universitaria di Bologna, Hypertension and Cardiovascular Risk Research Center, Medical and Surgical Sciences Dept., Alma Mater Studiorum University of Bologna, Bologna, ITALY Acquired data, critically reviewed the manuscript draft, and approved the submitted manuscript.

Teresa Valdés-Lacasa, Department of Gastroenterology, Hospital Universitario Infanta Cristina, Parla, SPAIN Acquired data, critically reviewed the manuscript draft, and approved the submitted manuscript.

Riccardo Vasapolli, Medical Department 2, University Hospital LMU Munich, Munich, GERMANY Acquired data, critically reviewed the manuscript draft, and approved the submitted manuscript.

Rosa M. Sáiz-Chumillas, Department of Gastroenterology, Hospital General de Almansa, Department of Gastroenterology, Complejo Hospitalario Universitario de Albacete, Almansa, SPAIN Acquired data, critically reviewed the manuscript draft, and approved the submitted manuscript.

Tamara Matysiak-Budnik, Hepato-Gastroenterology & Digestive Oncology Unit, University Hospital of Nantes, Nantes, FRANCE Acquired data, critically reviewed the manuscript draft, and approved the submitted manuscript.

Anna L. Pakhomova, V. I. Razumovsky Saratov state medical University, Ministry of health of Russia, Saratov, RUSSIA Acquired data, critically reviewed the manuscript draft, and approved the submitted manuscript.

Jorge Yebra Carmona, Department of Gastroenterology, Hospital Universitario de Móstoles, Móstoles, SPAIN Acquired data, critically reviewed the manuscript draft, and approved the submitted manuscript.

Diego Ledro Cano, Department of Gastroenterology, Clínica HLA Santa Isabel, Department of Gastroenterology, Hospital Universitario Virgen Macarena, Sevilla, SPAIN Acquired data, critically reviewed the manuscript draft, and approved the submitted manuscript.

Senador Moran Sanchez, Department of Gastroenterology, Servicio Murciano de Salud, Cartagena, SPAIN Acquired data, critically reviewed the manuscript draft, and approved the submitted manuscript.

Jan Bornschein Nuffield Department of Experimental Medicine, University of Oxford, Oxford, UNITED KINGDOM Acquired data, critically reviewed the manuscript draft, and approved the submitted manuscript.

Pilar Bernal Checa, Department of Gastroenterology, Hospital Universitario Miguel Servet, Instituto de Investigación Sanitaria de Aragón (IIS Aragón), Zaragoza, SPAIN Acquired data, critically reviewed the manuscript draft, and approved the submitted manuscript.

Antonio M. Caballero-Mateos, Department of Gastroenterology, Hospital Santa Ana, Precision Medicine, Instituto de Investigación Biosanitaria de Granada, Granada, SPAIN Acquired data, critically reviewed the manuscript draft, and approved the submitted manuscript.

Leticia Gimeno Pitarch, Department of Gastroenterology, Hospital General Universitario de Castellón, Castellón, SPAIN Acquired data, critically reviewed the manuscript draft, and approved the submitted manuscript.

Ángela Martínez Herreros, Department of Gastroenterology, Hospital Universitario San Pedro, Logroño, SPAIN Acquired data, critically reviewed the manuscript draft, and approved the submitted manuscript.

Nerea Alonso-Bilbao, Department of Gastroenterology, Hospital Universitario San Pedro, Logroño, SPAIN Acquired data, critically reviewed the manuscript draft, and approved the submitted manuscript.

Patricia Sanz-Segura, Department of Gastroenterology, Hospital Royo Villanova, Zaragoza, SPAIN Acquired data, critically reviewed the manuscript draft, and approved the submitted manuscript.

Jose Xavier Segarra Ortega, Department of Gastroenterology, Hospital Universitario de Salamanca, Grupo de Investigación Salmantino en Aparato Digestivo (GISAD), Instituto de Investigación Biomédica de Salamanca, Salamanca, SPAIN Acquired data, critically reviewed the manuscript draft, and approved the submitted manuscript.

Raquel García-Sánchez, Department of Gastroenterology, Hospital Universitario Infanta Leonor, Madrid, SPAIN Acquired data, critically reviewed the manuscript draft, and approved the submitted manuscript.

Alexander Link, Friedrich-Alexander-Universität Erlangen-Nürnberg (FAU), Medizincampus Oberfranken, Klinikum Bayreuth GmbH, Bayreuth, GERMANY Acquired data, critically reviewed the manuscript draft, and approved the submitted manuscript.

Jesus M. Gonzalez-Santiago, Department of Gastroenterology, Complejo Asistencial Universitario de Salamanca, Instituto de Investigación Biomédica de Salamanca (IBSAL), Centro de Investigación Biomédica en Red de Enfermedades Hepáticas y Digestivas (CIBERehd), Salamanca, SPAIN Acquired data, critically reviewed the manuscript draft, and approved the submitted manuscript.

Laura Larrey Ruiz, Department of Gastroenterology, Hospital Universitario Doctor Peset, Valencia, SPAIN Acquired data, critically reviewed the manuscript draft, and approved the submitted manuscript.

Rodrigo Garcés-Durán, Department of Gastroenterology and Hepatology, Université catholique de Louvain, Cliniques Universitaires Saint-Luc, Brussels, BELGIUM Acquired data, critically reviewed the manuscript draft, and approved the submitted manuscript.
