# Supplementary material for: Lack of Usefulness of Literature‐Based Helicobacter pylori Resistance Data for Predicting First‐Line Empirical Treatment Effectiveness in the European Registry on H. pylori Management (Hp‐EuReg)
Source: Helicobacter. 2026 Nov 5;31(6):e70169. doi: 10.1111/hel.70169 (PMC13735993; doi:10.1111/hel.70169)
Supplement: Supplementary file 5 [file HEL-31-e70169-s004.docx]

**Table S5.** Discriminatory performance of the general prediction models.

|  | **AUC (95% CI)** | **Threshold** | **Accuracy** | **Sensitivity** | **Specificity** |
| --- | --- | --- | --- | --- | --- |
| **Base model** | 0.68 (0.67-0.69) | 0.87 | 0.69 | 0.71 | 0.55 |
| **Model excluding country**  **and year** | 0.67 (0.65-0.68) | 0.89 | 0.61 | 0.61 | 0.64 |
| **Model including resistances** | 0.67 (0.66-0.68) | 0.88 | 0.66 | 0.67 | 0.58 |

Receiver operating characteristic (ROC) curve analysis was used to assess model discrimination. The area under the curve (AUC) with 95% confidence intervals (CI) was calculated using DeLong’s method. The optimal probability threshold was determined using the Youden index. Accuracy, sensitivity, and specificity were derived from confusion matrices after dichotomizing predicted probabilities according to the optimal cutoff. CI, confidence interval; AUC, area under the curve.
