# Supplementary material for: Lack of Usefulness of Literature‐Based Helicobacter pylori Resistance Data for Predicting First‐Line Empirical Treatment Effectiveness in the European Registry on H. pylori Management (Hp‐EuReg)
Source: Helicobacter. 2026 Nov 5;31(6):e70169. doi: 10.1111/hel.70169 (PMC13735993; doi:10.1111/hel.70169)
Supplement: Supplementary file 7 [file HEL-31-e70169-s006.docx]

**Table S4.** Variance inflation factor values for covariates included in the multivariate logistic regression model with antibiotic resistance variables.

|  | **VIF [95% CI]** | | **Adjusted VIF** | |
| --- | --- | --- | --- | --- |
| **Treatment** | 11.39 [11.10, 11.69] | 3.12 [3.05, 3.19] | 1.16 | 1.07 |
| **Sex** | 1.01 [1.00, 1.05] | 1.00 [1.00, 1.09] | 1.00 | 1.00 |
| **Age** | 1.02 [1.01, 1.04] | 1.01 [1.01, 1.04] | 1.01 | 1.01 |
| **PPI dose** | 1.43 [1.40, 1.45] | 1.30 [1.28, 1.32] | 1.09 | 1.07 |
| **Compliance** | 1.02 [1.01, 1.04] | 1.01 [1.00, 1.04] | 1.01 | 1.01 |
| **Length (days)** | 3.11 [3.04, 3.18] | 2.06 [2.02, 2.10] | 1.33 | 1.20 |
| **Country** | 1145.93 [1115.17, 1177.55] | - | 1.34 | - |
| **Year** | 16.16 [15.74, 16.59] | - | 1.17 | - |
| **Resistance to clarithromycin (current year)** | 66.54 [64.77, 68.36] | 3.56 [3.48, 3.65] | 8.16 | 1.89 |
| **Resistance to levofloxacin (current year)** | 17.60 [17.14, 18.07] | 2.95 [2.88, 3.02] | 4.20 | 1.72 |
| **Resistance to metronidazole (current year)** | 15.30 [14.91, 15.71] | 1.64 [1.61, 1.67] | 3.91 | 1.28 |

CI, confidence interval; PPI, proton-pump inhibitor; VIF, variance inflation factor.
