# Supplementary material for: Lack of Usefulness of Literature‐Based Helicobacter pylori Resistance Data for Predicting First‐Line Empirical Treatment Effectiveness in the European Registry on H. pylori Management (Hp‐EuReg)
Source: Helicobacter. 2026 Nov 5;31(6):e70169. doi: 10.1111/hel.70169 (PMC13735993; doi:10.1111/hel.70169)
Supplement: Supplementary file 8 [file HEL-31-e70169-s007.docx]

**Table S2.** Univariate and multivariate logistic regression analyses of predictors for eradication success in the modified intention-to-treat population.

| **Dependent variable: mITT** | | **Failure**  **(N=3835)** | **Success**  **(N=33786)** | **OR (95%CI, *P*)**  **[Univariate]** | **OR (95%CI, *P*)**  **[Multivariate]** |  |
| --- | --- | --- | --- | --- | --- | --- |
| **Treatment** | Sc-BQT | 604 (15.75%) | 604 (15.75%) |  |  | |
|  | Triple-CA | 1330 (34.68%) | 1330 (34.68%) | 0.41 (0.37-0.45, *P*<10^-3^) | 0.29 (0.25-0.33, *P*<10^-3^) | |
|  | Triple-CM | 211 (5.50%) | 211 (5.50%) | 0.26 (0.22-0.31, *P*<10^-3^) | 0.17 (0.13-0.21, *P*<10^-3^) | |
|  | Triple-AM | 45 (1.17%) | 45 (1.17%) | 0.25 (0.17-0.35, *P*<10^-3^) | 0.24 (0.17-0.36, *P*<10^-3^) | |
|  | Triple-AL | 73 (1.90%) | 73 (1.90%) | 0.32 (0.24-0.42, *P*<10^-3^) | 0.25 (0.19-0.34, *P*<10^-3^) | |
|  | Conco-CAT/M | 858 (22.37%) | 858 (22.37%) | 0.63 (0.56-0.70, *P*<10^-3^) | 0.58 (0.51-0.67, *P*<10^-3^) | |
|  | Seq-CAT/M | 240 (6.26%) | 240 (6.26%) | 0.67 (0.57-0.78, *P*<10^-3^) | 0.45 (0.36-0.55, *P*<10^-3^) | |
|  | Quad-CAB | 422 (11.00%) | 422 (11.00%) | 0.97 (0.85-1.10, *P*=0.61) | 0.65 (0.54-0.78, *P*<10^-3^) | |
|  | Quad-MTcB | 52 (1.36%) | 52 (1.36%) | 0.85 (0.63-1.14, *P*=0.28) | 0.69 (0.50-0.95, *P*=0.02) | |
| **Country** | Spain | 297 (7.74%) | 297 (7.74%) |  |  | |
|  | Belgium | 429 (11.19%) | 429 (11.19%) | 0.92 (0.78-1.08, *P*=0.31) | 0.88 (0.75-1.04, *P*=0.15) | |
|  | Bulgaria | 373 (9.73%) | 373 (9.73%) | 1.00 (0.85-1.18, *P*=0.99) | 0.87 (0.74-1.04, *P*=0.12) | |
|  | France | 412 (10.74%) | 412 (10.74%) | 1.16 (0.99-1.36, *P*=0.07) | 0.78 (0.66-0.93, *P*=5x10^-3^) | |
|  | Germany | 344 (8.97%) | 344 (8.97%) | 1.26 (1.07-1.49, *P*=0.01) | 0.71 (0.59-0.85, *P*<10^-3^) | |
|  | Israel | 273 (7.12%) | 273 (7.12%) | 1.66 (1.39-1.97, *P*<10^-3^) | 0.83 (0.69-1.01, *P*=0.06) | |
|  | Italy | 302 (7.87%) | 302 (7.87%) | 1.37 (1.16-1.63, *P*<10^-3^) | 0.74 (0.61-0.89, *P*=2x10^-3^) | |
|  | Poland | 327 (8.53%) | 327 (8.53%) | 1.09 (0.92-1.28, *P*=0.34) | 0.57 (0.48-0.69, *P*<10^-3^) | |
|  | Portugal | 349 (9.10%) | 349 (9.10%) | 1.48 (1.25-1.74, *P*<10^-3^) | 0.77 (0.64-0.93, *P*=7x10^-3^) | |
|  | Russia | 284 (7.41%) | 284 (7.41%) | 1.97 (1.66-2.33, *P*<10^-3^) | 0.96 (0.79-1.17, *P*=0.69) | |
|  | Slovenia | 297 (7.74%) | 297 (7.74%) | 1.90 (1.60-2.25, *P*<10^-3^) | 0.91 (0.75-1.10, *P*=0.31) | |
|  | The Netherlands | 148 (3.86%) | 148 (3.86%) | 2.48 (2.01-3.04, *P*<10^-3^) | 1.18 (0.94-1.48, *P*=0.15) | |
|  | United Kingdom | 2041 (53.22%) | 2041 (53.22%) |  |  | |
| **Year** | 2013 | 10 (0.26%) | 10 (0.26%) | 0.62 (0.32-1.23, *P*=0.17) | 1.24 (0.62-2.47, *P*=0.54) | |
|  | 2014 | 3 (0.08%) | 3 (0.08%) | 0.42 (0.12-1.52, *P*=0.19) | 0.40 (0.10-1.52, *P*=0.18) | |
|  | 2015 | 14 (0.37%) | 14 (0.37%) | 0.96 (0.55-1.67, *P*=0.88) | 1.02 (0.57-1.82, *P*=0.95) | |
|  | 2016 | 23 (0.60%) | 23 (0.60%) | 2.06 (1.35-3.15, *P*<10^-3^) | 3.93 (2.54-6.10, *P*<10^-3^) | |
|  | 2017 | 29 (0.76%) | 29 (0.76%) | 0.64 (0.43-0.95, *P*=0.03) | 0.89 (0.58-1.36, *P*=0.59) | |
|  | 2018 | 306 (7.98%) | 306 (7.98%) | 1.33 (1.17-1.51, *P*<10^-3^) | 1.72 (1.43-2.05, *P*<10^-3^) | |
|  | 2019 | 51 (1.33%) | 51 (1.33%) | 0.94 (0.70-1.26, *P*=0.68) | 0.80 (0.58-1.12, *P*=0.196) | |
|  | 2020 | 87 (2.27%) | 87 (2.27%) | 0.87 (0.70-1.10, *P*=0.24) | 0.94 (0.74-1.19, *P*=0.62) | |
|  | 2021 | 821 (21.41%) | 821 (21.41%) | 1.04 (0.96-1.14, *P*=0.34) | 1.21 (1.07-1.37, *P*=2x10^-3^) | |
|  | 2022 | 336 (8.76%) | 336 (8.76%) | 1.02 (0.90-1.15, *P*=0.74) | 2.55 (2.13-3.05, *P*<10^-3^) | |
|  | 2023 | 7 (0.18%) | 7 (0.18%) | 1.73 (0.80-3.73, *P*=0.16) | 5.04 (2.31-11.00, *P*<10^-3^) | |
|  | 2024 | 107 (2.79%) | 107 (2.79%) | 0.29 (0.23-0.36, *P*<10^-3^) | 1.01 (0.76-1.35, *P*=0.94) | |
| **Sex** | Female | 2493 (65.01%) | 2493 (65.01%) |  |  | |
|  | Male | 1342 (34.99%) | 1342 (34.99%) | 1.20 (1.12-1.28, *P*<10^-3^) | 1.15 (1.07-1.24, *P*<10^-3^) | |
| **Age** | Mean ± SD | 50.36 ± 14.88 | 50.36 ± 14.88 | 1.00 (1.00-1.00, *P*=0.07) | 1.00 (1.00-1.00, *P*=0.17) | |
| **PPI potency of acid inhibition** | Low-dose | 2031 (52.96%) | 2031 (52.96%) |  |  | |
|  | Standard-dose | 994 (25.92%) | 994 (25.92%) | 1.57 (1.45-1.70, *P*<10^-3^) | 1.51 (1.38-1.65, *P*<10^-3^) | |
|  | High-dose | 810 (21.12%) | 810 (21.12%) | 2.03 (1.87-2.21, *P*<10^-3^) | 1.66 (1.51-1.83, *P*<10^-3^) | |
| **Compliance** | No (< 90% drug intake) | 238 (6.21%) | 238 (6.21%) |  |  | |
|  | Yes (>90% drug intake) | 3597 (93.79%) | 3597 (93.79%) | 6.41 (5.42-7.59, *P*<10^-3^) | 6.73 (5.64-8.03, *P*<10^-3^) | |
| **Length (days)** | 7 days | 474 (12.36%) | 474 (12.36%) |  |  | |
|  | 10 days | 1914 (49.91%) | 1914 (49.91%) | 2.20 (1.97-2.45, *P*<10^-3^) | 1.47 (1.24-1.74, *P*<10^-3^) | |
|  | 14 days | 1447 (37.73%) | 1447 (37.73%) | 2.52 (2.25-2.82, *P*<10^-3^) | 1.70 (1.42-2.03, *P*<10^-3^) | |

Continuous variables are presented as mean (standard deviation, SD) and categorical variables as n (%) stratified by outcome. Results are expressed as Odds Ratios (OR) with 95% confidence intervals (CI) and *P* for each variable. Univariate models were fitted individually for each covariate and the multivariate model included treatment regimen, country, year of prescription, sex, age, proton pump inhibitor (PPI) potency, treatment duration, and compliance. Reference categories were: Sc-BQT (single-capsule bismuth quadruple therapy) for regimen, Spain for country, 2013 for year, female for gender, low PPI potency for acid inhibition, and adequate adherence for compliance. N=37,659. PPI dose: Low (4.5-27 mg), Intermediate (32-40 mg), High (54-128 mg). Compliance: Yes (>90% drug intake), No (<90% drug intake). CI, confidence interval; Conco-CAT/M, concomitant clarithromycin-amoxicillin-triple therapy; mITT, modified intention-to-treat; OR, Odds Ratio; PPI, proton-pump inhibitor; Quad-CAB, quadruple clarithromycin-amoxicillin-bismuth therapy; Quad-MTcB, quadruple metronidazole-tetracycline-bismuth therapy; Sc-BQT, single-capsule bismuth quadruple therapy; Seq-CAT/M, sequential clarithromycin-amoxicillin-metronidazole therapy; Triple-AL, amoxicillin-levofloxacin triple therapy; Triple-AM, amoxicillin-metronidazole triple therapy; Triple-CA, clarithromycin-amoxicillin triple therapy; Triple-CM, clarithromycin-metronidazole triple therapy.
