# Supplementary material for: Lack of Usefulness of Literature‐Based Helicobacter pylori Resistance Data for Predicting First‐Line Empirical Treatment Effectiveness in the European Registry on H. pylori Management (Hp‐EuReg)
Source: Helicobacter. 2026 Nov 5;31(6):e70169. doi: 10.1111/hel.70169 (PMC13735993; doi:10.1111/hel.70169)
Supplement: Supplementary file 9 [file HEL-31-e70169-s008.docx]

**Table S7.** Bootstrapped multivariate logistic regression models of predictors of eradication success population, stratified by treatment regimen.

|  | **Previous year resistances by treatment, 10,000 Bootstrap replicates stratified by mITT**  **OR (95%CI), *P* [Bootstrapped Multivariate]** | | | | | |
| --- | --- | --- | --- | --- | --- | --- |
|  |  |  |  |  |  |  |
| **Dependent variable: mITT** | **Sc-BQT** | **Triple-CA** | **Triple-CM** | **Conco-CAT/M** | **Seq-CAT/M** | **Quad-CAB** |
| **Gender**  [Male] | 0.86(0.71-1.03),*P*=0.10 | 1.24(1.07-1.43),*P*<10^-3^ | 0.85(0.44-1.69),*P*=0.64 | 1.16(0.98-1.37),*P*=0.07 | 2.15(1.52-2.95),*P*<10^-3^ | 0.38(0.02-4.15),*P*=0.21 |
| **Age** | 1.00(0.99-1.00),*P*=0.23 | 1.01(1.00-1.01),*P*<10^-3^ | 1.01(0.99-1.03),*P*=0.23 | 1.00(1.00-1.01),*P*=0.52 | 1.01(1.00-1.02),*P*=0.03 | 0.97(0.73-1.29),*P*=0.87 |
| **PPI potency of acid inhibition** [Standard] | 1.74(1.37-2.17),*P*<10^-3^ | 1.75(1.47-2.07),*P*<10^-3^ | 3.19(1.30-6.71),*P*<10^-3^ | 1.23(1.00-1.50),*P*=0.04 | 1.42(0.02-42.19),*P*=0.47 | 1.00(0.99-1.01),*P*=0.50 |
| **PPI potency of acid inhibition**  [High] | 2.07(1.61-2.63),*P*<10^-3^ | 2.26(1.76-2.84),*P*<10^-3^ | 2.34(0.83-5.14),*P*=0.05 | 1.94(1.57-2.37),*P*<10^-3^ | 1.89(1.29-2.69),*P*<10^-3^ | 2.09(1.38-3.18),*P*<10^-3^ |
| **Compliance**  [Yes (>90% intake)] | 11.49(7.72-16.97),*P*<10^-3^ | 6.70(4.13-10.57),*P*<10^-3^ | - | 4.87(3.49-6.83),*P*<10^-3^ | 3.69(1.61-8.77),*P*=0.01 | 1.72(1.09-2.80),*P*=0.03 |
| **Regimen days**  [10] | 0.00(0.00-0.00),*P*<10^-3^ | 1.04(0.86-1.26),*P*=0.70 | 0.86(0.35-2.13),*P*=0.73 | - | - | 7.66(3.77-15.71),*P*<10^-3^ |
| **Regimen days**  [14] | 0.00(0.00-0.00),*P*=0.01 | 1.29(1.03-1.62),*P*=0.03 | 1.05(0.37-2.93),*P*=0.93 | 0.96(0.81-1.14),*P*=0.67 | - | 3.66(0.36-45.91),*P*=0.07 |
| **Resistance to clarithromycin (last year)** | - | 1.00(0.99-1.01),*P*=0.66 | 0.98(0.95-1.02),*P*=0.29 | 1.02(1.00-1.03),*P*=0.01 | 1.01(0.99-1.03),*P*=0.22 | 3.15(0.32^-3^9.92),*P*=0.09 |
| **Resistance to levofloxacin (last year)** | - | - | - | - | - | - |
| **Resistance to metronidazole (last year)** | 1.00(0.99-1.01),*P*=0.98 | - | 1.00(0.96-1.04),*P*=0.86 | 0.99(0.98-1.01),*P*=0.58 | 1.01(0.98-1.03),*P*=0.60 | - |

Models were adjusted for sex, age, proton pump inhibitor (PPI) potency, compliance, regimen duration, and resistance rates for the previous year (clarithromycin, levofloxacin, metronidazole) where applicable. Results are expressed as odds ratios (OR) with 95% confidence intervals (CI) and P, based on 10,000 bootstrap replicates stratified by outcome in the mITT population. Values are shown separately for Sc-BQT (n= 5,852; Failure n= 434; Success n= 5,418), Triple-CA (n= 2,938; Failure n= 496; Success n= 2,442), Triple-CM (n= 83; Failure n= 39; Success n= 44), Conco-CAT/M (n= 3,892; Failure n= 435; Success n= 3,457), Seq-CAT/M (n= 1,049; Failure n= 103; Success n= 946), Quad-CAB (n= 1718; Failure n= 126; Success n= 1,592), and Quad-MTcB (n= 104; Failure n= 12; Success n= 92). Missing estimates (-) indicate covariates not applicable to that regimen due lack of n. PPI dose: Low (4.5-27 mg), Intermediate (32-40 mg), High (54-128 mg). Compliance: Yes (>90% drug intake), No (<90% drug intake). CI, confidence interval; Conco-CAT/M, concomitant clarithromycin-amoxicillin-triple therapy; mITT, modified intention-to-treat; OR, Odds Ratio; PPI, proton-pump inhibitor; Quad-CAB, quadruple clarithromycin-amoxicillin-bismuth therapy; Sc-BQT, single-capsule bismuth quadruple therapy; Seq-CAT/M, sequential clarithromycin-amoxicillin-metronidazole therapy; Triple-CA, clarithromycin-amoxicillin triple therapy; Triple-CM, clarithromycin-metronidazole triple therapy.
