# Supplementary material for: Lack of Usefulness of Literature‐Based Helicobacter pylori Resistance Data for Predicting First‐Line Empirical Treatment Effectiveness in the European Registry on H. pylori Management (Hp‐EuReg)
Source: Helicobacter. 2026 Nov 5;31(6):e70169. doi: 10.1111/hel.70169 (PMC13735993; doi:10.1111/hel.70169)
Supplement: Supplementary file 10 [file HEL-31-e70169-s009.docx]

**Table S1.** Spearman correlation coefficients between antibiotic resistance rates and eradication success on the modified intention-to-treat population across treatment regimens.

| **Sc-BQT** | | | |
| --- | --- | --- | --- |
| **Resistance** | **n** | ***ρ*** | ***P*** |
| Resistance to metronidazole (current year) | 20 | 0.02 | 0.94 |
| Resistance to metronidazole (previous year) | 25 | 0.25 | 0.24 |
| Resistance to metronidazole (current + previous 4 years) | 41 | 0.01 | 0.93 |
| **Triple-CA** | | | |
| **Resistance** | **n** | ***ρ*** | ***P*** |
| Resistance to clarithromycin (current year) | 58 | -0.04 | 0.74 |
| Resistance to clarithromycin (previous year) | 61 | -0.08 | 0.56 |
| Resistance to clarithromycin (current + previous 4 years) | 93 | -0.12 | 0.25 |
| **Triple-CM** | | | |
| **Resistance** | **n** | ***ρ*** | ***P*** |
| Resistance to clarithromycin (current year) | 34 | -0.18 | 0.30 |
| Resistance to metronidazole (current year) | 30 | -0.10 | 0.59 |
| Resistance to clarithromycin (previous year) | 36 | -0.15 | 0.39 |
| Resistance to metronidazole (previous year) | 33 | -0.32 | 0.07 |
| Resistance to clarithromycin (current + previous 4 years) | 56 | -0.28 | 0.04 |
| Resistance to metronidazole (current + previous 4 years) | 51 | -0.29 | 0.04 |
| **Triple-AM** | | | |
| **Resistance** | **n** | ***ρ*** | ***P*** |
| Resistance to metronidazole (current year) | 19 | -0.44 | 0.06 |
| Resistance to metronidazole (previous year) | 20 | -0.52 | 0.02 |
| Resistance to metronidazole (current + previous 4 years) | 36 | -0.31 | 0.06 |
| **Triple-AL** | | | |
| **Resistance** | **n** | ***ρ*** | ***P*** |
| Resistance to levofloxacin (current year) | 33 | -0.29 | 0.10 |
| Resistance to levofloxacin (previous year) | 35 | -0.14 | 0.41 |
| Resistance to levofloxacin (current + previous 4 years) | 50 | -0.05 | 0.73 |
| **Conco-CAT/M** | | | |
| **Resistance** | **n** | ***ρ*** | ***P*** |
| Resistance to clarithromycin (current year) | 34 | 0.10 | 0.57 |
| Resistance to metronidazole (current year) | 32 | -0.09 | 0.64 |
| Resistance to clarithromycin (previous year) | 38 | 0.25 | 0.14 |
| Resistance to metronidazole (previous year) | 37 | -0.19 | 0.26 |
| Resistance to clarithromycin (current + previous 4 years) | 56 | 0.05 | 0.70 |
| Resistance to metronidazole (current + previous 4 years) | 53 | -0.12 | 0.40 |
| **Seq-CAT/M** | | | |
| **Resistance** | **n** | ***ρ*** | ***P*** |
| Resistance to clarithromycin (current year) | 30 | 0.09 | 0.65 |
| Resistance to metronidazole (current year) | 25 | -0.05 | 0.82 |
| Resistance to clarithromycin (previous year) | 29 | -0.06 | 0.77 |
| Resistance to metronidazole (previous year) | 28 | -0.03 | 0.89 |
| Resistance to clarithromycin (current + previous 4 years) | 44 | -0.06 | 0.72 |
| Resistance to metronidazole (current + previous 4 years) | 41 | -0.09 | 0.58 |
| **Quad-CAB** | | | |
| **Resistance** | **n** | ***ρ*** | ***P*** |
| Resistance to clarithromycin (current year) | 15 | 0.36 | 0.19 |
| Resistance to clarithromycin (previous year) | 16 | 0.01 | 0.97 |
| Resistance to clarithromycin (current + previous 4 years) | 23 | 0.11 | 0.61 |
| **Quad-MTcB** | | | |
| **Resistance** | **n** | ***ρ*** | ***P*** |
| Resistance to metronidazole (current year) | 23 | -0.10 | 0.66 |
| Resistance to metronidazole (previous year) | 23 | -0.30 | 0.17 |
| Resistance to metronidazole (current + previous 4 years) | 35 | -0.35 | 0.04 |

Correlations were calculated per country-year, using resistance rates from the current year, the previous year, or current + four previous years. For each regimen and resistance variable, the table reports the number of country-year observations (n), the Spearman correlation coefficient (*ρ*), and the associated *P.* Conco-CAT/M, concomitant clarithromycin-amoxicillin-triple therapy; Quad-CAB, quadruple clarithromycin-amoxicillin-bismuth therapy; Quad-MTcB, quadruple metronidazole-tetracycline-bismuth therapy; Sc-BQT, single-capsule bismuth quadruple therapy; Seq-CAT/M, sequential clarithromycin-amoxicillin-metronidazole therapy; Triple-AL, amoxicillin-levofloxacin triple therapy; Triple-AM, amoxicillin-metronidazole triple therapy; Triple-CA, clarithromycin-amoxicillin triple therapy; Triple-CM, clarithromycin-metronidazole triple therapy.
