# Supplementary material for: Lack of Usefulness of Literature‐Based Helicobacter pylori Resistance Data for Predicting First‐Line Empirical Treatment Effectiveness in the European Registry on H. pylori Management (Hp‐EuReg)
Source: Helicobacter. 2026 Nov 5;31(6):e70169. doi: 10.1111/hel.70169 (PMC13735993; doi:10.1111/hel.70169)
Supplement: Supplementary file 11 [file HEL-31-e70169-s010.docx]

**Table S8.** Bootstrapped multivariate logistic regression models of predictors of eradication success population, stratified by treatment regimen.

|  | **Previous year resistances by treatment, 10,000 Bootstrap replicates stratified by mITT**  **OR (95%CI), *P* [Bootstrapped Multivariate]** | | | | | |
| --- | --- | --- | --- | --- | --- | --- |
|  |  |  |  |  |  |  |
| **Dependent variable: mITT** | **Sc-BQT** | **Triple-CA** | **Triple-CM** | **Conco-CAT/M** | **Seq-CAT/M** | **Quad-CAB** |
| **Gender**  [Male] | 0.84(0.70-1.00),*P*=0.05 | 1.30(1.14-1.48),*P*<10^-3^ | 0.98(0.60-1.61),*P*=0.94 | 1.16(1.00-1.35),*P*=0.05 | 2.18(1.57-2.96),*P*<10^-3^ | 0.99(0.79-1.24),*P*=0.96 |
| **Age** | 1.00(0.99-1.00),*P*=0.51 | 1.01(1.00-1.01),*P*<10^-3^ | 1.00(0.99-1.02),*P*=0.58 | 1.00(1.00-1.01),*P*=0.38 | 1.01(1.00-1.02),*P*=0.10 | 0.99(0.99-1.00),*P*=0.15 |
| **PPI potency of acid inhibition**  [Standard] | 1.67(1.32-2.08),*P*<10^-3^ | 1.51(1.30-1.75),*P*<10^-3^ | 3.43(1.51-7.14),*P*<10^-3^ | 1.16(0.97-1.39),*P*=0.09 | 1.91(0.30-9.06),*P*=0.13 | 1.74(1.27-2.41),*P*<10^-3^ |
| **PPI potency of acid inhibition**  [High] | 1.67(1.33-2.07),*P*<10^-3^ | 2.18(1.77-2.66),*P*<10^-3^ | 2.53(1.37-4.36),*P*<10^-3^ | 1.67(1.38-1.99),*P*<10^-3^ | 1.63(1.16-2.20),*P*<10^-3^ | 1.99(1.39-2.86),*P*<10^-3^ |
| **Compliance**  [Yes (>90% intake)] | 11.02(7.61-15.87),*P*<10^-3^ | 7.21(4.65-11.09),*P*<10^-3^ | - | 6.00(4.45-8.12),*P*<10^-3^ | 3.67(1.65-8.54),*P*=0.01 | 6.56(3.61-12.08),*P*<10^-3^ |
| **Regimen days**  [10] | 0.00(0.00-0.00),*P*<10^-3^ | 0.97(0.81-1.15),*P*=0.73 | 0.39(0.22-0.73),*P*<10^-3^ | 2.14(0.08-114.63),*P*=0.26 | - | 2.34(0.72-9.19),*P*=0.11 |
| **Regimen days**  [14] | 0.00(0.00-0.00),*P*<10^-3^ | 1.17(0.96-1.42),*P*=0.11 | 0.59(0.34-1.08),*P*=0.08 | 2.55(0.10-136.09),*P*=0.20 | 0.24(0.04-1.20),*P*=0.04 | 2.98(0.93-11.56),*P*=0.05 |
| **Resistance to clarithromycin (current + 4 previous years)** | - | 1.00(0.99-1.00),*P*=0.68 | 0.98(0.95-1.02),*P*=0.34 | 1.00(0.99-1.01),*P*=0.91 | 1.01(1.00-1.03),*P*=0.12 | 0.95(0.93-0.98),*P*<10^-3^ |
| **Resistance to levofloxacin (current + 4 previous years)** | - | - | - | - | - | - |
| **Resistance to metronidazole (current + 4 previous years)** | 0.99(0.98-1.01),*P*=0.49 | - | 0.97(0.95-1.01),*P*=0.10 | 1.00(0.99-1.02),*P*=0.60 | 0.98(0.95-1.01),*P*=0.26 | - |

Models were adjusted for sex, age, proton pump inhibitor (PPI) potency, compliance, regimen duration, and resistance rates for the current + 4 previous years (clarithromycin, levofloxacin, metronidazole) where applicable. Results are expressed as odds ratios (OR) with 95% confidence intervals (CI) and *P*, based on 10,000 bootstrap replicates stratified by outcome in the mITT population. Values are shown separately for Sc-BQT (n= 6,924; Failure n= 483; Success n= 6,441), Triple-CA (n= 5,124; Failure n= 820; Success n= 4,304), Triple-CM (n= 333; Failure n= 76; Success n=257), Conco-CAT/M (n= 5,412; Failure n= 561; Success n= 4,851), Seq-CAT/M (n= 1292; Failure n= 127; Success n= 1,165), Quad-CAB (n= 3.656; Failure n= 231; Success n= 3425), and Quad-MTcB (n= 423; Failure n= 49; Successs n= 374). Missing estimates (-) indicate covariates not applicable to that regimen due lack of n. PPI dose: Low (4.5-27 mg), Intermediate (32-40 mg), High (54-128 mg). Compliance: Yes (>90% drug intake), No (<90% drug intake). CI, confidence interval; Conco-CAT/M, concomitant clarithromycin-amoxicillin-triple therapy; mITT, modified intention-to-treat; OR, Odds Ratio; PPI, proton-pump inhibitor; Quad-CAB, quadruple clarithromycin-amoxicillin-bismuth therapy; Sc-BQT, single-capsule bismuth quadruple therapy; Seq-CAT/M, sequential clarithromycin-amoxicillin-metronidazole therapy; Triple-CA, clarithromycin-amoxicillin triple therapy; Triple-CM, clarithromycin-metronidazole triple therapy.
