# Supplementary material for: Lack of Usefulness of Literature‐Based Helicobacter pylori Resistance Data for Predicting First‐Line Empirical Treatment Effectiveness in the European Registry on H. pylori Management (Hp‐EuReg)
Source: Helicobacter. 2026 Nov 5;31(6):e70169. doi: 10.1111/hel.70169 (PMC13735993; doi:10.1111/hel.70169)
Supplement: Supplementary file 12 [file HEL-31-e70169-s011.docx]

**Table S9.** Performance metrics of Random Forest models classifying modified intention-to-treat outcomes.

|  | **Crosss-Validation** | | | **Hold-out performance** | | |
| --- | --- | --- | --- | --- | --- | --- |
| **Model** | **Mean PCC (%)** | **Mean Kappa** | **Median OOB PCC** | **Accuracy** | **Sensitivity** | **Specificity** |
| **Current year resistances model** | 65.86 | 0.14 | 65.78 | 0.67 | 0.69 | 0.54 |
| **Previous year resistances model** | 67.70 | 0.14 | 67.44 | 0.69 | 0.70 | 0.56 |
| **Last 5 years resistances model** | 66.14 | 0.13 | 66.26 | 0.67 | 0.68 | 0.59 |

Summary of Random Forest classification performance for the current-year, previous-year, and last-five-years resistance models. Metrics include mean percent correctly classified (Mean PCC %), mean Cohen’s Kappa, median out-of-bag (OOB) PCC, and hold-out test performance measures (accuracy, Kappa, sensitivity, and specificity). OOB, out-of-bag; PCC, Probability of Correct Classification.
